# High-*Q* microresonators unveil quantum rare events

**DOI:** 10.1126/sciadv.aed7827

**Published:** 2026-07-08

**Authors:** Sricharan Raghavan-Chitra, Arghadip Koner, Joel Yuen-Zhou

**Affiliations:** Department of Chemistry and Biochemistry, University of California San Diego, La Jolla, CA 92093, USA.

## Abstract

Classical linear optics posits that at sufficiently low intensities, light propagation in dielectric media is governed solely by their linear susceptibilities. Here, we demonstrate a departure from this paradigm in high–quality (high-*Q*) microresonators, where prolonged photon confinement enables rare quantum electrodynamical (QED) events, mediated by the quantum vacuum, to embed distinctive Raman signatures of the coupled analyte into the resonator’s linear transmission spectrum despite their absence from linear susceptibility. We further show that increasing the amount of adsorbed analyte sample amplifies these Raman fingerprints well above typical noise floors, rendering them experimentally accessible with state-of-the-art photonic architectures and detection schemes. This weak-coupling cavity QED effect offers unique routes to harness extended photon lifetimes and constrained geometries for leveraging vacuum fluctuations in next-generation photonic technologies for chemical and biological sensing and high-precision optical spectroscopy.

## Teaser

Material fingerprints, invisible to classical linear optics and mediated by the quantum vacuum, emerge in high-*Q* microresonators.

## INTRODUCTION

Propagation of weak-intensity light through dielectric media is typically considered a classical optics affair (*1*, *2*). The electric field amplitudes of transmitted, reflected, and absorbed light can be accurately described using the standard framework based on the linear permittivity of the material, $\epsilon^{\left(1\right)}\left(r,\omega \right)=\epsilon_{0}\left[1+\chi^{\left(1\right)}\left(r,\omega \right)\right]$, where $\chi^{\left(1\right)}\left(r,\omega \right)$ represents its linear susceptibility, and $\epsilon_{0}$ is the permittivity of free space (*3*, *4*). For example, light propagation through a multilayered dielectric medium can be accurately described using transfer matrix methods (*5*). One may, of course, decide to care about the photon statistics of light, upon which the correct formalism is quantum linear optics, where linear permittivities are still the only needed material input (*6*, *7*). Here, we investigate a light phenomenon that does not rely on measurements of quantum statistics or entanglement of the photons (*8*, *9*), yet the conventional framework of classical linear optics proves insufficient to fully describe certain scenarios of its linear propagation. In particular, we show that the (quantum) vacuum imprints unexpected and unusually useful information about the dielectric medium that is not captured by $\epsilon^{\left(1\right)}\left(r,\omega \right)$ and manifests already at the electric field amplitude level.

**Fig. 1.**
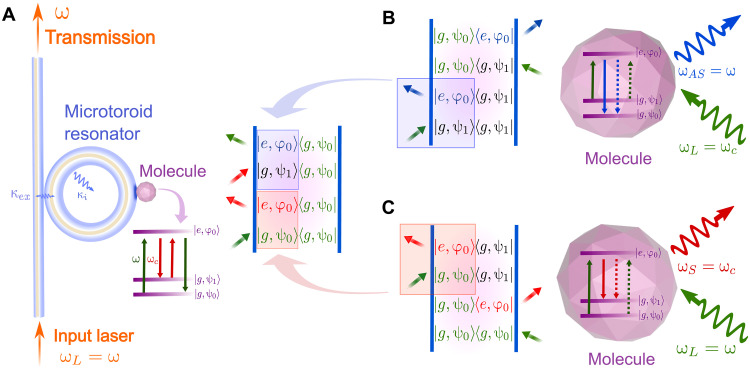
Schematic illustration of vacuum-mediated Raman processes in the linear optics of a microresonator system, highlighting the Stokes and anti-Stokes components. (**A**) A high-*Q* microtoroid resonator at frequency $\omega_{c}$ is evanescently coupled to a single molecule and probed with an incident laser at frequency ω, introduced via an optical fiber through evanescent coupling. The key phenomenon we reveal is that contrary to classical linear optics predictions, a molecule’s Raman vibrational fingerprints directly manifest in linear transmission, T(ω), of the microtoroid, provided that the cavity lifetime is sufficiently long. The adjacent ladder and DSFDs illustrate a cavity Raman process, where Stokes scattering populates the cavity vacuum with a field that subsequently drives the anti-Stokes transition. Notably, this mechanism solely involves quantum coherences, unlike the Purcell effect. Moreover, distinct from conventional Raman, it neither induces vibrational heating or cooling nor results in the up- or downconversion of photons. Instead, the process operates within the linear regime, introducing additional absorption channels at the Raman frequencies. This vacuum-mediated process cannot occur outside the single-mode cavity, as the Stokes field becomes irreversibly dispersed among the numerous modes of the free-field electromagnetic continuum. In contrast, panels (**B**) and (**C**) denote conventional anti-Stokes and Stokes Raman processes that occur when an incident laser photon at frequency $\omega_{L}$ inelastically scatters to $\omega_{\mathrm{AS}}$ and $\omega_{S}$ by extracting and leaving behind vibrational energy from the molecule, respectively. This leads to vibrational cooling (heating) as the population is transferred to the vibrational ground (excited) state. Being an inherently nonlinear process, this upconverts (downconverts) the laser photon, unlike in the linear regime of the microtoroid resonator. The corresponding DSFD (*12*) and ladder diagrams (*80*) illustrate the underlying mechanism. Solid and dotted lines in the ladder diagrams indicate the ket- and bra-side interactions in the DSFDs.

The scenario of interest is depicted in Fig. 1. A single molecule is deposited on the surface of a high–quality (high-*Q*) microtoroid resonator, which is, in turn, coupled to an optical fiber. This setup has become a standard molecular sensing tool in the past decade (*10*): Regardless of the coherence properties of the light (*11*), its transmission through the fiber acknowledges the presence of the single molecule via a tiny phase shift induced by the molecular permittivity $\chi^{\left(1\right)}\left(\omega \right)$ (*12*). The main feature that makes toroidal microresonators suitable for this formidable task is the unusually long photon lifetime: The ultrahigh *Q* factors achievable through advanced microfabrication techniques trap the photon for $Q=10^{3}\;\text{to}\;10^{10}$ round trips, amplifying the weak perturbations produced by the single molecule (*13*). More generally, the extraordinary photon lifetimes achievable in these resonators have found application across various fields like enhancing weak signals in gravitational wave detection (*14*), atmospheric particle tracking (*15*), and biosensing (*16*), among other areas. This longevity should also enable the detection of unlikely processes that nonetheless manifest if given enough time. Of our particular interest are rare events mediated by the quantum vacuum, such as that depicted in the energy-level diagram of Fig. 1. As the light with frequency ω passes through the microresonator, it can act as a pump inducing a Stokes Raman scattering event, modifying the vacuum with a red-shifted field at frequency $\omega_{c}$ while leaving a vibrational coherence at $\omega -\omega_{c}$ in the molecule. However, because of the high *Q* of the microresonator, there is a finite probability that this red-shifted field will be repurposed to act as a second pump, thereby regenerating the vacuum and simultaneously inducing an anti-Stokes Raman scattering event. The output light is at the same frequency ω as the incoming light; its amplitude is proportional to the amplitude of the latter, both features highlighting the linear optical nature of the phenomenon. However, the novelty is that contrary to the classical optics paradigm, it cannot be understood using the linear susceptibility, $\chi^{\left(1\right)}\left(\omega \right)$, alone (Raman scattering events can only be described with higher-order susceptibilities involving more than two transition dipole moments) (*17*). The resolution to this apparent conundrum is that two of the dipole interactions are mediated by the vacuum.

The phenomenon described above is undetectable outside of these highly controlled photonic environments (*10*). Owing to the three-dimensional nature of the electromagnetic continuum, it is entropically unlikely that the emitted Stokes Raman field will be reabsorbed by the molecule in free space. This is not an issue in the microtoroid resonator where the discreteness of its mode structure (*10*), as well as the longevity of the trapped photon (*18*), “forces” the Stokes field to interact again with the molecule. This constitutes a weak-coupling cavity quantum electrodynamical phenomenon that is qualitatively different from established phenomena such as the Purcell effect (*19*, *20*); as opposed to the latter, it benefits from a low density of photon modes, which is precisely what the three-dimensional confinement of the microtoroid resonator offers.

With this preamble, this work leverages confined geometries and prolonged photon lifetimes to capture rare events, introducing a previously unidentified paradigm for microresonator applications and paving the way for innovative approaches in chemical and biosensing as well as advancements in Raman technology.

## RESULTS

### Linear response of microtoroid coupled to a single molecule

High-*Q* microresonators with three-dimensional confinement, such as microspheres and microtoroids, are emerging as a frontier in sensing, with a growing effort toward operation across a diverse spectral range. When coupled to a molecule, this optical confinement in three dimensions leads to a discrete mode structure instrumental to the quantum vacuum–mediated Raman, while the high-*Q* nature of the resonator allows enhanced sensitivity to resolve the subtle spectral features in the linear spectra. Here, we establish a theoretical framework that rigorously captures both the optical mode of a microtoroid resonator and the quantum dynamics of the molecule. By using an input-output formalism, we compute the frequency-resolved transmission spectrum for arbitrary molecular complexity, revealing molecular Raman signatures mediated through quantum vacuum and emerging at the spectral tails.

The interaction between an optical toroidal microresonator and a single molecule can be effectively described by a reduced model when the frequency range of interest is narrower than the free spectral range (FSR). Under these conditions, the Hamiltonian for the setup is given as (*18*)

$$H=\sum_{\alpha =a,b}H_{\text{ph},\alpha }+H_{I}+H_{\text{mol}}+V$$ (1)

where, in natural units, $H_{\text{ph},\alpha }=\omega_{c}\alpha^{†}\alpha$ governs the two degenerate counterpropagating optical modes arising because of the intrinsic symmetry of the resonator; α=a (*b*) denotes the annihilation operator for the clockwise (counterclockwise) mode. Scattering processes induced by structural imperfections in the resonator couple these modes via $H_{I}=\beta \left(a^{†}b+b^{†}a\right)$, with β denoting the coupling strength. Furthermore, these optical modes couple to a molecule via an evanescent interaction, which, under the rotating wave approximation (RWA), is described by

$$V=g\left(a^{†}+b^{†}\right)\sigma +h.c$$ (2)

where σ denotes the lowering operator associated with the electronic transition of the molecule. The parameter $g=\sqrt{\frac{\omega_{c}}{2\epsilon_{0}V_{m}}}$ represents the single-molecule light-matter coupling strength, with $V_{m}$ being the effective mode volume and $\epsilon_{0}$ being the permittivity of free space.

Under the RWA, the interaction V preserves the total excitation number. As a result, in the linear-response regime considered here, the dynamics are confined to the first excitation manifold. This truncation is standard in molecular absorption calculations (*12*) even in the absence of a resonator and remains well justified for all coupling regimes explored in this work, which do not enter the ultrastrong-coupling regime. The corresponding molecular Hamiltonian is therefore

$$H_{\text{mol}}=\sum_{n}\omega_{g,n}|g,\psi_{n}\rangle \langle g,\psi_{n}|+\sum_{m}\omega_{e,m}|e,\varphi_{m}\rangle \langle e,\varphi_{m}|$$ (3)

where $|g,\psi_{n}\rangle$ and $|e,\varphi_{m}\rangle$ represent the vibronic states in the ground and electronically excited manifolds, with corresponding energies $\omega_{g,n}$ and $\omega_{e,m}$, respectively.

For high-*Q* cavities, the reflection, R(ω), is inherently minimal [see section S1 where we cite (*18*, *21*–*25*)], making transmission, T(ω), the dominant experimentally accessible signal (*26*). Conservation of photon flux enables us to express the frequency-resolved transmission in terms of the absorption, T(ω)=1−A(ω) (*25*). We will primarily present absorption spectra throughout this work to facilitate direct comparisons with bare molecular spectra.

The absorption spectrum, A(ω), of the microresonator coupled to a single molecule can be rigorously obtained using input-output theory [see sections S1 and S2 where we cite (*12*, *18*, *21*–*25*)] in terms of the photon Green’s function (*18*, *23*). Given that only the symmetric superposition of the optical modes of the resonator interacts with the molecule, it is convenient to define the annihilation operators $X=\frac{a+b}{\sqrt{2}}$ and $Y=\frac{a-b}{\sqrt{2}}$, corresponding to the symmetric and antisymmetric modes, respectively. This leads to the linear absorption of the microtoroid-molecule system being decomposable into distinct contributions (symmetric and antisymmetric)

$$A\left(\omega \right)=\sum_{\alpha =X,Y}A^{\alpha }\left(\omega \right)$$ (4)

with

$$A^{\alpha }\left(\omega \right)=-\kappa_{\text{ex}}ℑ\left[D^{\alpha }\left(\omega \right)\right]-\frac{\kappa_{\text{ex}}^{2}}{2}{|D^{\alpha }\left(\omega \right)|}^{2}$$ (5)

Here, $D^{\alpha }\left(\omega \right)=\langle 1_{\text{ph}}^{\alpha };g,\psi_{0}|G\left(\omega \right)|1_{\text{ph}}^{\alpha };g,\psi_{0}\rangle$ is the matrix element of the total Green’s function, $G\left(\omega \right)=\frac{1}{\omega -H+i0^{+}}$, corresponding to the quantum state $|1_{\text{ph}}^{\alpha };g,\psi_{0}\rangle$ with a photon in the α mode of the cavity and the molecule in the global ground state (*25*, *27*, *28*). Furthermore, the field decay rate for the resonator normal modes is $\kappa =\kappa_{i}+\kappa_{\text{ex}}$, where $\kappa_{i}$ represents intrinsic losses and $\kappa_{\text{ex}}$ describes extrinsic loss due to (adjustable) couplings of the modes to the fiber coupler (*18*).

Given that the antisymmetric optical mode remains decoupled from the molecular bath, its absorption component

$$A^{Y}\left(\omega \right)=\frac{\kappa_{\text{ex}}\kappa_{i}}{{\left[\omega -\left(\omega_{c}-\beta \right)\right]}^{2}+\frac{\kappa^{2}}{4}}$$ (6)

is dictated solely by the resonator’s intrinsic dissipation, producing a characteristic Lorentzian profile. In contrast, the absorption of the symmetric mode, $A^{X}\left(\omega \right)$, encodes modifications in the Lorentzian line shape arising from molecular interactions, imprinting a spectral fingerprint of the molecule within the response of the resonator (*29*).

**Fig. 2.**
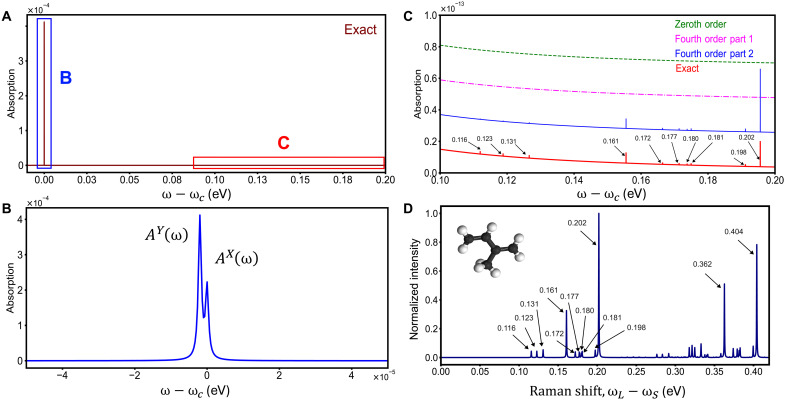
Cavity vacuum–mediated Raman processes in isoprene encode the information of the bare Raman spectrum. (**A**) Frequency-resolved absorption spectrum of a microresonator coupled to a single isoprene molecule. The exact spectrum is plotted in maroon with $\kappa_{\text{ex}}=\gamma_{e}=\gamma_{\text{vib}}=10^{-6}$ eV, and $\kappa_{i}\ll \kappa_{\text{ex}}$ (*18*) is taken to be 10^−10^ eV. (**B**) Blue-highlighted region of (A) showing the lifting of degeneracy in the microresonator modes, which leads to symmetric and antisymmetric combinations. (**C**) Red-highlighted region of (A) showing the spectral structure near the cavity resonance at $\omega =\omega_{c}$. The exact spectrum (red) is the only curve shown on the correct absolute vertical scale. For visual clarity, the remaining contributions are displayed with artificial vertical offsets: “Fourth order part 2” is plotted with an offset of 0.22 × 10^−13^ eV, “fourth order part 1” with 0.44 × 10^−13^ eV, and the “zeroth order” term with 0.66 × 10^−13^ eV. The zeroth-order term (green) in Eq. 7 reproduces the bare cavity spectrum. Including up to the first part (pink) of the fourth-order term in the Dyson expansion accounts only for Rayleigh features, encoding only the linear susceptibility $\chi^{\left(1\right)}\left(\omega \right)$ of the molecule and thus remaining within the paradigm of classical linear optics. In contrast, including the second part of the fourth-order term (blue) unveils additional subtle peaks around the cavity resonance: signatures of quantum vacuum–mediated Stokes-anti-Stokes Raman processes that introduce additional absorption channels at frequencies detuned from the cavity resonance by the Raman shifts. (**D**) Raman spectrum of bare isoprene (*81*), where the *y* axis represents the normalized scattered intensity and the *x* axis corresponds to the Raman shift, defined as the frequency difference between incident and scattered light.

Contrary to $A^{Y}\left(\omega \right)$, the computation of $A^{X}\left(\omega \right)$ is nontrivial because of the inherent complexity of the molecule (*25*). In Fig. 2, we present the numerically computed $A^{X}\left(\omega \right)$ using the exact photon Green’s function $D^{X}\left(\omega \right)$ [sections S3 and S4 where we cite (*12*, *17*, *30*, *31*)]. For this calculation, we used isoprene, a well-established benchmark for studying linear and nonlinear optical responses in condensed-phase systems because of its well-characterized electronic structure and biological relevance, making it an ideal candidate for validating theoretical and experimental methods in spectroscopy and photophysics (*32*–*34*). Its electronic transition occurs at a characteristic frequency of $\omega_{0-0}=6.026$ eV with an oscillator strength of 0.5523, while its vibrational energy landscape has been parametrized with 10 displaced harmonic oscillators (table S2) (*32*). Furthermore, this molecule is evanescently coupled to the m=27th mode of the microtoroidal resonator, which is red detuned by Δ=0.3 eV relative to $\omega_{0-0}$. Under these conditions, the light-matter coupling strength is determined to be $g=1.63\times 10^{-4}$ eV [see section S7 where we cite (*35*, *36*)] on the basis of an approximated mode volume (*37*). This parameter set corresponds to the weak-coupling regime, as the ratio g/Δ remains much smaller than unity. Intriguingly, an additional set of peaks emerges near the cavity resonance in the absorption spectrum (Fig. 2). These features are distinct from Purcell-enhanced Raman scattering, cavity-enhanced Raman spectroscopy, and other familiar cavity-induced weak-coupling effects (*19*, *20*), which describe nonlinear inelastic scattering processes governed primarily by the cavity-modified photonic density of states. By contrast, the present peaks appear directly in the linear absorption response of the coupled resonator-molecule system and can be captured by a Dyson expansion of the photon Green’s function, $D^{\alpha }\left(\omega \right)$, in the light-matter coupling *V*.

The photon Green’s function up to the fourth-order term in the Dyson series

$$D^{X}\left(\omega \right)=D_{\left(0\right)}^{X}\left(\omega \right)+D_{\left(2\right)}^{X}\left(\omega \right)g^{2}+D_{\left(4\right)}^{X}\left(\omega \right)g^{4}+O\left(g^{6}\right)$$ (7)

is sufficient to explain the appearance of the additional peaks in Fig. 2 near the cavity resonance. Higher-order contributions merely rescale the intensity of existing features rather than introducing any additional ones [see section S4 and S5 where we cite (*12*, *17*, *30*, *31*)]. Given that $D^{\alpha }\left(\omega \right)$ is a diagonal component of the total Green’s function G(ω), all odd-order terms in the Dyson expansion vanish, leaving only even-order corrections (*12*).

In the absence of molecules, the zeroth-order cavity response, $D_{\left(0\right)}^{X}\left(\omega \right)=1/\left(\omega -\omega_{c}+i\frac{\kappa }{2}\right)$, accounts for the Lorentzian profile of the bare microtoroid resonator (*18*). With the introduction of light-matter coupling, the second-order contribution is $D_{\left(2\right)}^{X}\left(\omega \right)=-g^{2}{\left[D_{\left(0\right)}^{X}\left(\omega \right)\right]}^{2}\chi^{\left(1\right)}\left(\omega \right)$; here, $\chi^{\left(1\right)}\left(\omega \right)=-{\text{lim}}_{\gamma \to 0^{+}}\sum_{n=0}\frac{{|\langle \psi_{0}|\varphi_{n}\rangle |}^{2}}{\omega -\omega_{e,n}+i\frac{\gamma }{2}}$ is the linear susceptibility of the molecule representing the cavity-mediated Rayleigh-type scattering processes through which energy is dissipated into the molecular bath (*12*, *30*). Here, $\gamma \to 0^{+}$ ensures the causality of the Green’s function and can be chosen arbitrarily small in simulations as the molecular complexity is explicitly captured by introducing sufficient vibrational modes (*38*). This second-order term is captured in classical optics and reveals how molecular interactions perturb the Lorentzian profile of the bare resonator, introducing an additional decay channel driven by dipole coupling to the molecules.

Last, the fourth-order term $D_{\left(4\right)}^{X}\left(\omega \right)=g^{4}{\left[D_{\left(0\right)}^{X}\left(\omega \right)\right]}^{3}{\left[\chi^{\left(1\right)}\left(\omega \right)\right]}^{2}+R_{\text{vib}}\left(\omega \right)$ modifies the cavity line shape in two distinct ways. The first contribution incorporates the classical optical effects of higher-order Rayleigh scattering in the spectral response, whereas the second term

$$R_{\text{vib}}\left(\omega \right)=g^{4}{\left[D_{\left(0\right)}^{X}\left(\omega \right)\right]}^{2}\sum_{m=1}^{M_{g}}\frac{\langle \psi_{0}|G_{\text{ex}}\left(\omega \right)|\psi_{m}\rangle \langle \psi_{m}|G_{\text{ex}}\left(\omega \right)|\psi_{0}\rangle }{\omega -\left(\omega_{c}+\omega_{g,m}\right)+i\frac{\left(\kappa +\gamma_{\text{vib}}\right)}{2}}$$ (8)

is beyond classical optics and describes interactions involving the cavity photon and ground-state molecular vibrations of frequency $\omega_{g,m}$ with an associated decay rate $\gamma_{\text{vib}}$; here, $G_{\text{ex}}\left(\omega \right)=\sum_{m}\frac{|\varphi_{m}\rangle \langle \varphi_{m}|}{\left(\omega -\omega_{e,m}+i\frac{\gamma }{2}\right)}$ is the excited-state Green’s function of the molecule. These processes generate additional spectral features near the cavity resonance, $\omega_{c}$, as shown in Fig. 2, that are not captured by lower-order treatments. Equation 8 approximated near the cavity resonance, $R_{\text{vib}}\left(\omega \right)\approx \frac{1}{2\text{π}}g^{4}{\left[D_{\left(0\right)}^{X}\right]}^{2}S_{\text{Raman}}\left(\omega_{L}=\omega ,\omega_{S}=\omega_{c}\right)$, captures the same information as the Stokes Raman spectra in Fig. 2A, where

$$S_{\text{Raman}}\left(\omega_{L},\omega_{S}\right)=2\pi \sum_{m=1}^{\infty }{|\langle \psi_{0}|G_{\text{ex}}\left(\omega_{L}\right)|\psi_{m}\rangle |}^{2}\delta \left(\omega_{S}-\omega_{L}+\omega_{g,m}\right)$$ (9)

denotes the Kramers-Heisenberg-Dirac Stokes Raman cross section (*12*, *39*) with a laser of frequency $\omega_{L}$ undergoing inelastic scattering to leave behind a vibrationally hot molecule and a red-shifted Stokes field at $\omega_{S}=\omega_{L}-\omega_{g,m}$ (see Fig. 1).

To highlight the underlying mechanism, Eq. 8 can be reorganized into [section S4 where we cite (*12*, *17*, *30*, *31*)]

$$R_{\text{vib}}\left(\omega \right)\approx \frac{g^{4}}{2\pi }{\left[D_{\left(0\right)}^{X}\left(\omega \right)\right]}^{2}\sqrt{S_{\text{Raman}}\left(\omega_{L}=\omega ,\omega_{S}=\omega_{c}\right)}\sqrt{S_{\text{Raman}}^{C}\left(\omega_{L}=\omega_{c},\omega_{\mathrm{AS}}=\omega \right)}$$ (10)

where the conditional Kramers-Heisenberg-Dirac anti-Stokes Raman cross section

$$S_{\text{Raman}}^{C}\left(\omega_{L},\omega_{\mathrm{AS}}\right)=2\pi \sum_{m=1}^{\infty }{|\langle \psi_{m}|G_{\text{ex}}\left(\omega_{L}\right)|\psi_{0}\rangle |}^{2}\delta \left(\omega_{\mathrm{AS}}-\omega_{L}-\omega_{g,m}\right)$$ (11)

describes the inverse scenario where an incident photon of frequency $\omega_{L}$ inelastically scatters by extracting vibrational energy from the molecule, leaving behind a vibrationally cold molecule at the global ground state, while the scattered photon emerges blue-shifted at $\omega_{\mathrm{AS}}=\omega_{L}+\omega_{g,m}$, as illustrated in Fig. 1

Equation 10 represents the underlying vacuum-mediated Stokes and conditional anti-Stokes processes, as illustrated in Fig. 1, using the double-sided Feynman diagram (DSFD). The incoming laser field at frequency $\omega_{L}=\omega$ drives a Stokes scattering process, leaving behind a scattered field at the cavity resonance, $\omega_{S}=\omega_{c}$. This scattered field, in turn, serves as the incident field for a constrained anti-Stokes process at $\omega_{L}=\omega_{c}$, ultimately yielding a final scattered field at the original laser frequency, $\omega_{\mathrm{AS}}=\omega$. This previously unknown vacuum-mediated phenomenon opens additional absorption channels at frequencies detuned from the cavity resonance by the Raman shifts, thereby encoding Raman signatures directly within the linear optical response of the microtoroidal resonator. Although its microscopic origin involves virtual processes analogous to Raman scattering, the resulting signal remains strictly within the linear-response regime, given that it is detected at the probe frequency and scales linearly with the probe intensity.

The parameters chosen for Fig. 2 are not fully representative of realistic experimental conditions and are instead intended solely to demonstrate the existence of the phenomenon and its underlying mechanism in the simplified case of a single molecule coupled to the cavity. Under realistic conditions, the presence of multiple noise sources, including photon shot noise, which scales as $\propto \sqrt{N_{\text{ph}}}$ (*40*), and the intrinsic dark current of photodetectors, typically on the order of 10^5^ photons for silicon photodiodes at room temperature (*41*), poses substantial challenges for the experimental observation of these subtle spectral features. Given that their strength is on the order of 10^−13^ *N*_ph_, achieving a signal above the photon shot noise would require averaging over 10^26^ photons. Such an intense photon flux risks inducing spurious effects resulting from photothermal effects and nonlinear optical processes (*42*) and also exceeds the saturation thresholds of conventional detectors (*43*). In the following section, we introduce a detection scheme designed to enhance the signal-to-noise ratio by leveraging collective interactions for a molecular ensemble, thereby proportionally amplifying the detected signal and mitigating the limitations imposed by detector and shot noise.

### Detection scheme

Enhancements of absorption cross sections can be directly achieved by increasing the number of absorbers (*44*). In our system, this corresponds to increasing the number of absorbers evanescently coupled to the microtoroidal resonator. The upper limit for the surface density of such absorbers is $\rho_{\text{max}}=5\times 10^{14}{\text{cm}}^{-2}$ (*45*), which, given the estimated inner and outer diameters of the resonator [see section S8 where we cite (*45*)], translates to a maximum of 10^7^ molecules that can be evanescently coupled. However, an exact computation of the photon Green’s function for a resonator interacting with millions of molecules is an intractable problem. Consequently, a Dyson expansion approach provides the only feasible (and well justified) framework for evaluating the photon Green’s function in this regime (*25*, *27*).

For a microtoroid resonator resonantly coupled to 3 × 10^6^ isoprene molecules, a Dyson expansion of the photon Green’s function in the light-matter interaction *V* reveals the existence of two dynamical timescales: a timescale corresponding to the collective coupling $g\sqrt{N}$ that drives the Rayleigh scattering processes and a single-molecule timescale, 2π/g, corresponding to the sought-after Raman processes. To address this inherently multiscale problem, we incorporate the collective effects exactly via a self-consistent Dyson equation (section S6) while treating *g*-mediated single-molecule interactions perturbatively.

**Fig. 3.**
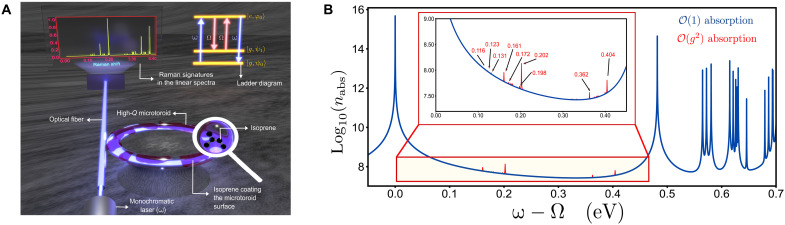
Proposed experimental protocol for enhanced Raman peak detection in the linear absorption spectra. (**A**) Schematic of the experimental protocol for Raman peak enhancement, where an ensemble of isoprene molecules is evanescently coupled to a microtoroid resonator (highlighted by the magnifying glass). An optical fiber delivers the laser, which couples evanescently to the resonator, thereby imprinting the molecular Raman signatures onto the absorption spectrum in the linear regime. Corresponding ladder diagram illustrating the energy-level transitions, Ω assuming the role that $\omega_{c}$ took in the single-molecule case. (**B**) The main plot presents the logarithm (base 10) of the number of absorbed photons, ${\text{log}}_{10}n_{\text{abs}}$, as a function of ω−Ω, where Ω corresponds to the lower peak of the absorption spectrum. The system parameters are set as Δ=0, $g=1.63\times 10^{-4}$ eV, $N=3\times 10^{6}$, $\kappa_{\text{ex}}=\gamma =3\times 10^{-5}$ eV, and $\gamma_{\text{vib}}=5\times 10^{-4}$ eV (considering $\omega_{c}=\omega_{0-0}$, this corresponds to $Q=2\times 10^{5}$). The inset highlights the second-order correction to the absorption, revealing distinct Raman signatures. Both the fundamental Raman band and its higher-order overtones emerge, suggesting that vacuum-mediated effects play a role in the observed vibrational features. Notably, the photon absorption remains well above the noise equivalent power (NEP = $10^{5}$), with $n_{\text{abs}}>10^{7}$.

As a result, the zeroth-order photon Green’s function is modified by the fast Rayleigh transitions

$$D_{\left(0\right)}^{X\left(N\right)}\left(\omega \right)=\frac{1}{\omega -\omega_{c}+i\frac{\kappa }{2}+N\chi^{\left(1\right)}\left(\omega \right)}$$ (12)

which naturally embeds the linear molecular susceptibility of the ensemble, capturing all orders of the collective Rayleigh processes [see section S9 where we cite (*46*, *47*)] (which are all the effects in classical linear optics). Beyond this, the second-order term in the expansion

$$\begin{array}{c} D_{\left(2\right)}^{X\left(N\right)}\left(\omega \right)\propto g^{2}{\left(g\sqrt{N}\right)}^{2}{\left[D_{\left(0\right)}^{X\left(N\right)}\left(\omega \right)\right]}^{2}\\ \sqrt{S_{\text{Raman}}\left(\omega_{L}=\omega ,\omega_{S}=\Omega \right)}\sqrt{S_{\text{Raman}}^{C}\left(\omega_{L}=\Omega ,\omega_{\mathrm{AS}}=\omega \right)} \end{array}$$ (13)

evaluated around the lower absorption peak, Ω (modified by collective interactions), can be expressed in terms of Stokes and constrained anti-Stokes Raman cross sections, akin to Eq. 10. However, two key distinctions emerge. First, in contrast to the single-molecule case, where the Stokes-scattered field, serving as the pump for the ensuing anti-Stokes transition, is generated at $\omega_{c}$, the corresponding field in this regime manifests at frequency Ω (see DSFD in Fig. 3 for visualization). This results in Raman signatures emerging along the spectral tails of the Lorentzian profile centered at Ω instead of $\omega_{c}$, as illustrated in Fig. 3. The resulting Raman peaks from the exact expression for $D_{\left(2\right)}^{X\left(N\right)}\left(\omega \right)$ reveal an enhancement of the signal strength, effectively overcoming noise contributions in the spectrum with a sufficient number of molecules. In addition, the bare Raman spectrum of condensed-phase isoprene in Fig. 2D shows overtone features with intensities comparable to the fundamental peaks, reflecting the relatively large Huang-Rhys factors associated with the dominant 0.161- and 0.202-eV vibrational modes (*32*). Because these Raman-type signatures appear as vibrational sidebands of the cavity-molecule absorption spectrum, the corresponding overtones are likewise visible in Fig. 3B.

The Raman signal now reaches around 10^8^ photons, exceeding the intrinsic dark current of photodetectors (*41*) while remaining below saturation (*43*), as only 10^16^ photons need to be integrated for shot noise–limited measurements. However, rapid detection by focusing $∼10^{16}$ photons/s into a micrometer-scale fiber demands precise alignment and may introduce optical damage or nonlinear photothermal effects (*42*). Alternatively, lowering flux and extending integration might reduce high-intensity issues but heighten susceptibility to low-frequency drifts (*48*) and 1/*f* noise (*49*). Nonetheless, $O\left(10^{-8}\right)$ absorption detection is well within reach using active stabilization (*50*), lock-in amplification (*51*), balanced detection (*52*), and modulation-demodulation techniques (*53*), while heterodyne techniques (*54*) and short repetitive scans shift signals (*55*) beyond 1/*f* noise. Leveraging these strategies enables the precise extraction of these vacuum-mediated effects from technical noise in state-of-the-art and emerging photonic architectures (*56*).

**Fig. 4.**
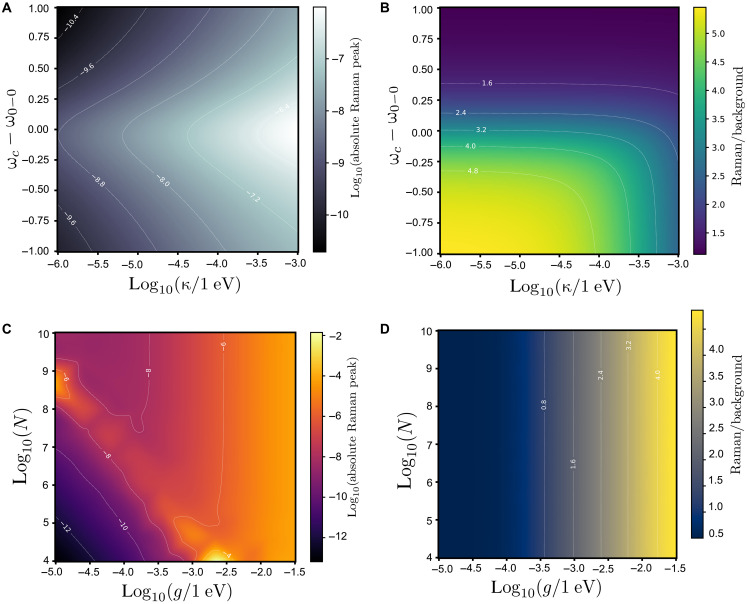
Dependence of absolute Raman peak height and its ratio to the Rayleigh background on key experimental parameters. Contour plots of (**A**) the absolute Raman peak height as a function of $\Delta =\omega_{c}-\omega_{0-0}$ and ${\text{log}}_{10}\left(\kappa /1\text{eV}\right)$ and (**B**) the ratio of Raman peak height to the Rayleigh background over the same parameter space with $g=1.63\times 10^{-4}$ eV, $N=3\times 10^{6}$, $\gamma =3\times 10^{-5}$ eV, and $\gamma_{\text{vib}}=5\times 10^{-4}$ eV for this simulation. (**C**) Absolute Raman peak height as a function of ${\text{log}}_{10}\left(g/1\text{eV}\right)$ and ${\text{log}}_{10}\left(N\right)$ and (**D**) ratio of Raman peak height to the Rayleigh background over the same parameter space with $\kappa_{\text{ex}}=\gamma =3\times 10^{-5}$ eV and $\gamma_{\text{vib}}=5\times 10^{-4}$ eV for this simulation. These simulations focus on the dominant Raman feature from the vibrational mode with frequency (ω_v_ = 0.202 eV) and Huang-Rhys factor (*S* = 1.57). Panels (A) and (B) indicate that *Q* factors in the range of 2 × 10^4^ to 2 × 10^6^ are optimal for maximizing both the signal strength and Raman/background ratio.

Moreover, given the multitude of parameters in Eq. 13, our detection scheme enables precise tuning to further enhance Raman peak visibility. In Fig. 4, we illustrate how the ratio of the Raman signal to its background, arising from Rayleigh Lorentzian tails, varies with the cavity detuning, Δ, and its linewidth, κ. As κ decreases, both the Rayleigh and Raman peaks become spectrally sharper, thereby improving this ratio. However, the trade-off is that the absolute Raman peak heights decrease in Fig. 4 as they are both filtered by and superimposed on the Rayleigh background. A similar dependence emerges upon tuning Δ away from resonance, given that detuning modifies the linewidth in a manner analogous to changes in κ [see section S6 where we cite (*12*, *17*, *30*, *31*)]. Together, Fig. 4 (A and B) demonstrates that *Q* factors between 2 × 10^4^ and 2 × 10^6^ provide the most favorable conditions for enhancing Raman signal strength while improving the Raman/background ratio.

In addition, in Fig. 4, we further consolidate our previous understanding into the roles of *g* or *N* in improving Raman peak visibility. As anticipated, increasing *N* raises the absolute Raman intensity, given that both the Rayleigh and Raman contributions are enhanced. In contrast, the signal-to-background ratio only improves with increasing *g*, consistent with the *g*^4^ scaling of the Raman response in Eq. 13.

We emphasize that the observability of these Raman sidebands near the cavity peak requires the light-matter coupling strength *g* to be comparable to (or greater than) κ, $\gamma_{e}$, and $\gamma_{\text{vib}}$. Here, the linewidths $\gamma_{e}$ and $\gamma_{\text{vib}}$ are introduced phenomenologically to account for coupling to both intramolecular and condensed-phase modes that are not explicitly described. This condition reflects the fact that the process illustrated in Fig. 1 relies on the coherent exchange of the Stokes field from the molecule to the cavity and back to the molecule before these decoherence channels become operative. Consequently, the relative scaling among the various decoherence rates does not by itself limit the phenomenon, except in the regime where they become much larger than *g*.

Notably, our model does not explicitly account for the inhomogeneous broadening of the molecular ensemble electronic transitions, as the Raman features near frequency Ω lie well outside the Gaussian envelope associated with the inhomogeneous component of the bare molecular absorption. This behavior is consistent with the polaron-decoupling regime, in which a sufficiently detuned probe is largely insensitive to the inhomogeneous distribution of molecular transition energies (*57*). In the regime of Fig. 3, where $g\sqrt{N}\approx 0.3$ eV exceeds the typical condensed-phase inhomogeneous broadening of isoprene (∼0.07 eV), we therefore expect the lower peak and its associated Raman-type sidebands to remain largely robust against energetic disorder.

In the present treatment, we do not explicitly resolve the effects of orientational averaging of the adsorbed molecules or the spatial structure of the cavity mode. Such effects are expected to enter primarily through a geometric renormalization (*58*) of collective light-matter coupling, i.e., as an effective prefactor multiplying $g\sqrt{N}$, rather than as an additional broadening mechanism. Consequently, while they may rescale the Rayleigh peak splitting and modify the Raman sideband amplitudes, they are not expected to qualitatively alter the visibility or interpretation of the Raman-type features discussed here.

## DISCUSSION

Our work explores an unprecedented frontier in photonic sensing and spectroscopy, where rare quantum vacuum fluctuations imprint Raman signatures in a linear optical response, rendering the signal independent of light’s coherence properties (*11*, *59*), and hence pave the way for cost-effective and simplified Raman technology. Owing to the off-resonant Raman scattering character of the phenomenon of interest, non-Condon (Herzberg-Teller) effects can also yield a substantial boost in the desired signal (*60*, *61*). The precise dependence of our signal on these contributions will be explored in future studies. Notably, unlike conventional nonlinear Raman techniques, our protocol inherently circumvents the common issue of fluorescence background by strictly prohibiting electronic population generation (*12*, *62*). Consequently, the inherently weak Raman signal remains unobscured and free from the masking effects of fluorescence. The appearance of the Raman signal in the linear spectra, however, introduces an inherent competition with classical optical absorption, a challenge that can be effectively mitigated by using off-resonant microresonators. Detuning from optical absorption also prevents deleterious photoinduced processes in the analyte from laser excitation. The parameter space explored in this work (e.g., FSR, mode volume, etc.) aligns with emerging microresonator technologies, suggesting the full utilization of the quantum vacuum effect with next-generation photonics (*63*–*65*). Nevertheless, state-of-the-art microresonators in the mid-infrared–to–ultraviolet regimes, with FSRs in the range of 5 to 10 meV and ultrahigh *Q* factors of 10^8^ to 10^10^, offer an ideal platform for terahertz Raman spectroscopy (*66*), substantially simplifying experimental protocols by reducing requirements for fluorescence suppression and Rayleigh filtering. The decrease in signal strength due to the increased mode volume in these mid-infrared resonators can be compensated by the enhancement from the increased surface area, allowing for the deposition of a larger ensemble of molecules [see Eq. 13 and section S6 where we cite (*12*, *17*, *30*, *31*)]. Beyond increasing the molecular ensemble, this platform offers a compelling opportunity to integrate emerging quantum light interferometry techniques, potentially amplifying both signal strength and sensitivity, which is especially relevant for detecting weak signals in low-frequency regimes (*67*–*69*).

From a broader perspective, harnessing the long photon lifetimes in high-*Q* microresonators to probe the cavity quantum electrodynamical rare events reported here is reminiscent of advances across diverse scientific fields, ranging from neutrino detection (Super-Kamiokande, IceCube) (*70*, *71*) and gravitational wave observation (LIGO, Virgo) (*72*) to rare decay experiments (*73*, *74*) and investigations of rare biochemical reactions (*75*), where extended observation times enhance sensitivity and facilitate the capture of elusive, low-probability events. In the ensemble protocol, in addition to the quantum vacuum–mediated third-order Raman susceptibility appearing as the leading-order quantum correction to the classical optics, additional features emerge from the higher-order quantum corrections that are not captured by any third-order nonlinear susceptibility (section S6) (*12*). These electrodynamical events are even rarer and demand emerging ultrahigh-*Q* microresonators to be observed (*66*, *76*, *77*). In addition, beyond the RWA, the weak counterrotating terms may enable access to higher manifolds and possibly hyper-Raman scattering in the linear spectra (*78*, *79*). Looking forward, this work proposes the ambitious goal of leveraging rare quantum vacuum–mediated events in high-*Q* resonators coupled to dielectric media, uncovering valuable dynamical information, and pushing the frontiers of advanced sensing and spectroscopic technology.

## Supplementary Materials

### This PDF file includes:

Sections S1 to S9 Figs. S1 to S3 Tables S1 and S2 References
