# Supplementary material for: High-Q microresonators unveil quantum rare events
Source: Sci Adv. 2026 Jul 8;12(28):eaed7827. doi: 10.1126/sciadv.aed7827 (PMC13344283; doi:10.1126/sciadv.aed7827)
Supplement: Supplementary file 1 — Sections S1 to S9 Figs. S1 to S3 Tables S1 and S2 References [file sciadv.aed7827_sm.pdf]

Science Advances
AAAS

Supplementary Materials for

High-*Q* microresonators unveil quantum rare events

Sricharan Raghavan-Chitra *et al.*

Corresponding author: Arghadip Koner, akoner@ucsd.edu; Joel Yuen-Zhou, joelyuen@ucsd.edu

Sci. Adv. **12**, eaed7827 (2026)
DOI: 10.1126/sciadv.aed7827

This PDF file includes:

Sections S1 to S9
Figs. S1 to S3
Tables S1 and S2
References

1 Input-output theory of a microtoroid resonator coupled to N molecules

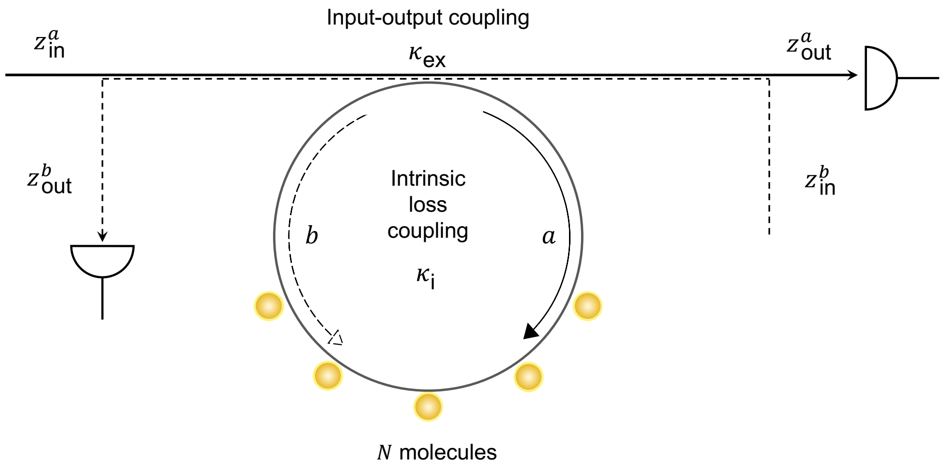

Figure S1: Schematic of microtoroid and fiber coupler

We derive formulas for linear spectroscopy of microtoroid cavity coupled to N molecules using input-output (IO) theory (*19, 22–25*). We couple this molecular cavity system (m-c-s) with z^a and z^b radiative continua via the photon mode,

$$H_{\text{total}} = H + \sum_{\alpha=a,b} H^{z^\alpha}, \tag{S1}$$

where,

$$H^{z^\alpha} = \hbar \int_0^\infty d\omega z^{\alpha\dagger}(\omega) z^\alpha(\omega)\omega + \hbar\sqrt{\frac{\kappa_{\text{ex}}}{2\pi}} \int_0^\infty d\omega a z^{\alpha\dagger}(\omega) + \text{h.c.} \tag{S2}$$

for $\alpha = a, b$ only feature Rotating Wave Approximation (RWA) terms and

$$[z^\alpha(\omega), z^{\alpha'}(\omega')] = [z^{\alpha\dagger}(\omega), z^{\alpha'\dagger}(\omega')] = 0, \tag{S3a}$$

$$[z^\alpha(\omega), z^{\alpha'\dagger}(\omega')] = \delta_{\alpha\alpha'}\delta(\omega - \omega'). \tag{S3b}$$

For convenience, we will now derive some results in the Heisenberg picture (corresponding to evolution with respect to H_{total}), with the corresponding operators labeled by the subscript H, e.g., $X_{\text{H}}(t) = e^{-iH_{\text{total}}(t-t_{\text{in}})/\hbar} X e^{iH_{\text{total}}(t-t_{\text{in}})/\hbar}$. Schrödinger picture operators will continue to be indicated

without an explicit subscript. The Equation of Motion (EoM) for the cavity photon corresponding to the symmetric mode is

$$\begin{aligned}\frac{\partial X_{\mathrm{H}}(t)}{\partial t} &= -\frac{i}{\hbar}\Big[X_{\mathrm{H}}(t), H\Big] \\ &= -i\big(\omega_{\mathrm{ph}}+\beta\big)X_{\mathrm{H}}(t) - \frac{\kappa_i}{2}X_{\mathrm{H}}(t) - i\sqrt{2}\sum_{i=1}^{N} g\,|g_i\rangle\langle e_i| - \frac{i}{\sqrt{2}}\sqrt{\frac{\kappa_{\mathrm{ex}}}{2\pi}}\int_0^\infty d\omega\Big[z^a_{\mathrm{H}}(\omega)(t) + z^b_{\mathrm{H}}(\omega)(t)\Big]\end{aligned} \tag{S4}$$

and the EoM for the $Y_{\mathrm{H}}(t)$ is

$$\begin{aligned}\frac{\partial Y_{\mathrm{H}}(t)}{\partial t} &= -\frac{i}{\hbar}\Big[Y_{\mathrm{H}}(t), H\Big] \\ &= -i\big(\omega_{\mathrm{ph}}-\beta\big)Y_{\mathrm{H}}(t) - \frac{\kappa_i}{2}Y_{\mathrm{H}}(t) - i\frac{1}{\sqrt{2}}\sqrt{\frac{\kappa_{\mathrm{ex}}}{2\pi}}\int_0^\infty d\omega\Big[z^a_{\mathrm{H}}(\omega)(t) - z^b_{\mathrm{H}}(\omega)(t)\Big].\end{aligned} \tag{S5}$$

Similarly, the corresponding EoM for the bath mode $z^a_{\mathrm{H}}(\omega)(t)$ is

$$\begin{aligned}\frac{\partial z^a_{\mathrm{H}}(\omega)(t)}{\partial t} &= -i\omega z^a_{\mathrm{H}}(\omega)(t) - i\sqrt{\frac{\kappa_{\mathrm{ex}}}{2\pi}}\frac{\big[X_{\mathrm{H}}(t)+Y_{\mathrm{H}}(t)\big]}{\sqrt{2}} \\ \Longrightarrow\quad \frac{\partial\big[z^a_{\mathrm{H}}(\omega)(t)e^{i\omega t}\big]}{\partial t} &= -i\sqrt{\frac{\kappa_{\mathrm{ex}}}{2\pi}}\frac{\big[X_{\mathrm{H}}(t)+Y_{\mathrm{H}}(t)\big]}{\sqrt{2}}e^{i\omega t}.\end{aligned} \tag{S6}$$

Defining $t_{\mathrm{in}} < t$ and $t_{\mathrm{out}} > t$, we can integrate Eq. S6 to obtain

$$z^a_{\mathrm{H}}(\omega)(t) = z^a_{\mathrm{H}}(\omega)(t_{\mathrm{in}})e^{-i\omega(t-t_{\mathrm{in}})} - \frac{i}{\sqrt{2}}\sqrt{\frac{\kappa_{\mathrm{ex}}}{2\pi}}\int_{t_{\mathrm{in}}}^{t} dt' X_{\mathrm{H}}(t')e^{-i\omega(t-t')} - \frac{i}{\sqrt{2}}\sqrt{\frac{\kappa_{\mathrm{ex}}}{2\pi}}\int_{t_{\mathrm{in}}}^{t} dt' Y_{\mathrm{H}}(t')e^{-i\omega(t-t')}, \tag{S7}$$

and

$$z^a_{\mathrm{H}}(\omega)(t) = z^a_{\mathrm{H}}(\omega)(t_{\mathrm{out}})e^{-i\omega(t-t_{\mathrm{out}})} + \frac{i}{\sqrt{2}}\sqrt{\frac{\kappa_{\mathrm{ex}}}{2\pi}}\int_{t}^{t_{\mathrm{out}}} dt' X_{\mathrm{H}}(t')e^{-i\omega(t-t')} + \frac{i}{\sqrt{2}}\sqrt{\frac{\kappa_{\mathrm{ex}}}{2\pi}}\int_{t}^{t_{\mathrm{out}}} dt' Y_{\mathrm{H}}(t')e^{-i\omega(t-t')}. \tag{S8}$$

Similarly, we can obtain expressions for $z^b_{\mathrm{H}}(\omega)(t)$:

$$z^b_{\mathrm{H}}(\omega)(t) = z^b_{\mathrm{H}}(\omega)(t_{\mathrm{in}})e^{-i\omega(t-t_{\mathrm{in}})} - \frac{i}{\sqrt{2}}\sqrt{\frac{\kappa_{\mathrm{ex}}}{2\pi}}\int_{t_{\mathrm{in}}}^{t} dt' X_{\mathrm{H}}(t')e^{-i\omega(t-t')} - \frac{i}{\sqrt{2}}\sqrt{\frac{\kappa_{\mathrm{ex}}}{2\pi}}\int_{t_{\mathrm{in}}}^{t} dt' Y_{\mathrm{H}}(t')e^{-i\omega(t-t')}, \tag{S9}$$

$$z^b_{\mathrm{H}}(\omega)(t) = z^b_{\mathrm{H}}(\omega)(t_{\mathrm{out}})e^{-i\omega(t-t_{\mathrm{out}})} + \frac{i}{\sqrt{2}}\sqrt{\frac{\kappa_{\mathrm{ex}}}{2\pi}}\int_{t}^{t_{\mathrm{out}}} dt' X_{\mathrm{H}}(t')e^{-i\omega(t-t')} + \frac{i}{\sqrt{2}}\sqrt{\frac{\kappa_{\mathrm{ex}}}{2\pi}}\int_{t}^{t_{\mathrm{out}}} dt' Y_{\mathrm{H}}(t')e^{-i\omega(t-t')}. \tag{S10}$$

Let us feed Eq. S7 & S9 into Eq. S4 & S5 and by approximating $\int_0^\infty d\omega' \approx \int_{-\infty}^\infty d\omega'$, we get,

$$\frac{\partial X_{\mathrm{H}}(t)}{\partial t} = -i\big(\omega_{\mathrm{ph}} + \beta\big)X_{\mathrm{H}}(t) - \frac{\kappa}{2}X_{\mathrm{H}}(t) + \sqrt{2}\sum_{i=1}^{N} g\,|g_i\rangle\langle e_i| - \sqrt{\kappa_{\mathrm{ex}}}\big[z^a_{\mathrm{in,H}}(t) + z^b_{\mathrm{in,H}}(t)\big], \tag{S11a}$$

$$\frac{\partial X_{\mathrm{H}}(t)}{\partial t} = -i\big(\omega_{\mathrm{ph}} + \beta\big)X_{\mathrm{H}}(t) - \frac{\kappa_{\mathrm{i}}}{2}X_{\mathrm{H}}(t) + \sqrt{2}\sum_{i=1}^{N} g\,|g_i\rangle\langle e_i| + \frac{\kappa_{\mathrm{ex}}}{2}X_{\mathrm{H}}(t) - \sqrt{\kappa_{\mathrm{ex}}}\big[z^a_{\mathrm{out,H}}(t) + z^b_{\mathrm{out,H}}(t)\big], \tag{S11b}$$

$$\frac{\partial Y_{\mathrm{H}}(t)}{\partial t} = -i\big(\omega_{\mathrm{ph}} - \beta\big)Y_{\mathrm{H}}(t) - \frac{\kappa}{2}Y_{\mathrm{H}}(t) - \sqrt{\kappa_{\mathrm{ex}}}\big[z^a_{\mathrm{in,H}}(t) - z^b_{\mathrm{in,H}}(t)\big], \tag{S11c}$$

$$\frac{\partial Y_{\mathrm{H}}(t)}{\partial t} = -i\big(\omega_{\mathrm{ph}} - \beta\big)Y_{\mathrm{H}}(t) - \frac{\kappa_{\mathrm{i}}}{2}Y_{\mathrm{H}}(t) + \frac{\kappa_{\mathrm{ex}}}{2}Y_{\mathrm{H}}(t) - \sqrt{\kappa_{\mathrm{ex}}}\big[z^a_{\mathrm{out,H}}(t) - z^b_{\mathrm{out,H}}(t)\big], \tag{S11d}$$

where $\kappa = \kappa_{\mathrm{i}} + \kappa_{\mathrm{ex}}$ and,

$$z^a_{\mathrm{in,H}}(t) = \frac{i}{\sqrt{2\pi}}\int_{-\infty}^{\infty} d\omega z^a_{\mathrm{H}}(\omega)(t_{\mathrm{in}})e^{-i\omega(t-t_{\mathrm{in}})}, \tag{S12a}$$

$$z^b_{\mathrm{in,H}}(t) = \frac{i}{\sqrt{2\pi}}\int_{-\infty}^{\infty} d\omega z^b_{\mathrm{H}}(\omega)(t_{\mathrm{in}})e^{-i\omega(t-t_{\mathrm{in}})}, \tag{S12b}$$

$$z^a_{\mathrm{out,H}}(t) = \frac{i}{\sqrt{2\pi}}\int_{-\infty}^{\infty} d\omega z^a_{\mathrm{H}}(\omega)(t_{\mathrm{out}})e^{-i\omega(t-t_{\mathrm{out}})}, \tag{S12c}$$

$$z^b_{\mathrm{out,H}}(t) = \frac{i}{\sqrt{2\pi}}\int_{-\infty}^{\infty} d\omega z^b_{\mathrm{H}}(\omega)(t_{\mathrm{out}})e^{-i\omega(t-t_{\mathrm{out}})}. \tag{S12d}$$

Using Eq. S11, we obtain the IO relations for the bath z^a,

$$z^a_{\mathrm{out,H}}(t) - z^a_{\mathrm{in,H}}(t) = \sqrt{\kappa_{\mathrm{ex}}}a_{\mathrm{H}}(t) \tag{S13}$$

and similarly, we can compute for bath z^b,

$$z^b_{\mathrm{out,H}}(t) - z^b_{\mathrm{in,H}}(t) = \sqrt{\kappa_{\mathrm{ex}}}b_{\mathrm{H}}(t) \tag{S14}$$

Assuming that the density matrix at $t = t_{\mathrm{in}}$ is a product state between the molecular microtoroidal cavity and the continua,

$$\rho_{\mathrm{total}}(t_{\mathrm{in}}) = \rho_{z^a}(t_{\mathrm{in}}) \otimes \rho(t_{\mathrm{in}}) \otimes \rho_{z^b}(t_{\mathrm{in}}), \tag{S15}$$

and that the driving occurs only from the z^a bath,

$$\langle z^a_{\mathrm{in,H}}(t)\rangle \neq 0 \tag{S16a}$$

$$\langle z^b_{\mathrm{in,H}}(t)\rangle = 0, \tag{S16b}$$

we get, after tracing over the continua,

$$\frac{\partial X_{\rm H}(t)}{\partial t} = -i\big(\omega_{\rm ph} + \beta\big)X_{\rm H}(t) - \frac{\kappa}{2}X_{\rm H}(t) + \sqrt{2}\sum_{i=1}^{N} g\,|g_i\rangle\,\langle e_i|\,(t) - \sqrt{\kappa_{\rm ex}}\langle z^a_{\rm in,H}(t)\rangle \tag{S17}$$

and

$$\frac{\partial Y_{\rm H}(t)}{\partial t} = -i\big(\omega_{\rm ph} - \beta\big)Y_{\rm H}(t) - \frac{\kappa}{2}Y_{\rm H}(t) - \sqrt{\kappa_{\rm ex}}\langle z^a_{\rm in,H}(t)\rangle \tag{S18}$$

Eq. S17 can be rewritten as

$$\frac{\partial X_{\rm H}(t)}{\partial t} = -\frac{i}{\hbar}\big[X_{\rm H}(t), \tilde{H}^X_{\rm H}(t)\big] \tag{S19}$$

which allows us to conclude that the effective time-dependent Hamiltonian (in the Schrodinger picture) governing the molecular microtoroid cavity system is

$$\tilde{H}^X(t) = H^{X'} + H^X_{\rm int}(t). \tag{S20}$$

In the absence of drive, the molecular microtoroidal cavity system obeys the effective non-Hermitian Hamiltonian,

$$H^{X'} = \hbar\Big[\omega_{\rm ph} + \beta - i\frac{\kappa}{2}\Big]X^\dagger X + \sqrt{2}\sum_{i=1}^{N}\hbar g X^\dagger\,|g_i\rangle\,\langle e_i| + \sqrt{2}\sum_{i=1}^{N}\hbar g X\,|e_i\rangle\,\langle g_i| \tag{S21}$$

while the time-dependent drive of the cavity due to light coupling from the bath z^a is

$$H^X_{\rm int}(t) = -i\hbar\sqrt{\kappa_{\rm ex}}\langle z^a_{\rm in,H}(t)\rangle X^\dagger + h.c. \tag{S22}$$

Similarly, we can use Eq. S18 and write the following set of equations that describes the effective Hamiltonian:

$$\frac{\partial Y_{\rm H}(t)}{\partial t} = -\frac{i}{\hbar}\big[Y_{\rm H}(t), \tilde{H}^Y_{\rm H}(t)\big], \tag{S23a}$$

$$\tilde{H}^Y_{\rm H}(t) = H^{Y'}_{\rm H} + H^Y_{\rm int,H}(t), \tag{S23b}$$

$$H^{Y'}_{\rm H} = \hbar\Big[\omega_{\rm ph} - \beta - i\frac{\kappa}{2}\Big]Y^\dagger Y, \tag{S23c}$$

$$H^Y_{\rm int,H}(t) = -i\hbar\sqrt{\kappa_{\rm ex}}\langle z^a_{\rm in,H}(t)\rangle Y^\dagger + h.c. \tag{S23d}$$

Equipped with this formalism, we are interested in computing the following spectroscopic observables:

$$
\begin{aligned}
T(\omega) &= \frac{|\langle z^a_{\text{out,H}}(\omega)\rangle|^2}{|\langle z^a_{\text{in,H}}(\omega)\rangle|^2} = \frac{|\langle z^a_{\text{in,H}}(\omega)\rangle + \sqrt{\kappa_{\text{ex}}}\langle a_{\text{H}}(\omega)\rangle|^2}{|\langle z^a_{\text{in,H}}(\omega)\rangle|^2} \\
&= \frac{|\langle z^a_{\text{in,H}}(\omega)\rangle + \frac{\sqrt{\kappa_{\text{ex}}}}{2}\Big[\langle X_{\text{H}}(\omega)\rangle + \langle Y_{\text{H}}(\omega)\rangle\Big]|^2}{|\langle z^a_{\text{in,H}}(\omega)\rangle|^2}, \qquad \text{(S24a)} \\
R(\omega) &= \frac{|\langle z^b_{\text{out,H}}(\omega)\rangle|^2}{|\langle z^a_{\text{in,H}}(\omega)\rangle|^2} = \frac{|\sqrt{\kappa_{\text{ex}}}\langle b_{\text{H}}(\omega)\rangle|^2}{|\langle z^a_{\text{in,H}}(\omega)\rangle|^2} \\
&= \frac{|\frac{\sqrt{\kappa_{\text{ex}}}}{2}\Big[\langle X_{\text{H}}(\omega)\rangle - \langle Y_{\text{H}}(\omega)\rangle\Big]|^2}{|\langle z^a_{\text{in,H}}(\omega)\rangle|^2}, \qquad \text{(S24b)} \\
A(\omega) &= 1 - T(\omega) - R(\omega) \qquad \text{(S24c)}
\end{aligned}
$$

where the traces above are carried out with respect to the initial state [Eq. S15], $\langle\cdot\rangle = \text{Tr}\big[\cdot\rho_{\text{total}}(t_{\text{in}})\big]$, and in particular, $\langle\alpha_{\text{H}}(\omega)\rangle = \text{Tr}\big[\alpha_{\text{H}}(\omega)\rho(t_{\text{in}})\big]$ with $\alpha = X, Y$ depends only on the initial state of the molecular microtoroidal cavity system. We have also used Eq. S16 and the Fourier transform convention, $f(\omega) = -i\int_{-\infty}^{\infty} dt e^{i\omega t} f(t)$. Equation S24 reveal that all the relevant spectroscopic observables can be obtained once $\langle X_{\text{H}}(\omega)\rangle$ and $\langle Y_{\text{H}}(\omega)\rangle$ are known.

2 Kubo linear response

Hereafter, we set $t_{\text{in}} = 0$. As H contains anharmonic terms, the evaluation of $\langle X(\omega)\rangle$ cannot be performed exactly. Hence, we carry out a Dyson expansion in H_{int} for each of the Heisenberg operators in Eq. S19 and solve for $X^{(n)}_{\text{H}}(t)$ up to lowest nonvanishing order n. (*26*)

Starting at zeroth-order, $O(H^0_{\text{int}})$,

$$
\frac{\partial X^{(0)}_{\text{H}}(t)}{\partial t} - \frac{i}{\hbar}\big[H^{X'}, X^{(0)}_{\text{H}}(t)\big] = 0 \qquad \text{(S25)}
$$

can be solved by

$$
X^{(0)}_{\text{H}}(t) = e^{iH^{X'}t/\hbar} X e^{-iH^{X'}t/\hbar}. \qquad \text{(S26)}
$$

Recall our assumption that the initial molecular microtoroidal cavity state is a product state between

photon and molecular degrees of freedom, $\rho(0) = \rho_{\text{ph}} \otimes \rho_{\text{mol}}$,

$$e^{-iH^{X'}t/\hbar}\rho(0) \overset{t\to\infty}{=} |0\rangle \langle\varphi_{\text{ph}}| \otimes \rho_{\text{mol}}, \tag{S27}$$

where $|\varphi_{\text{ph}}\rangle$ is a photonic state. Then

$$\langle X_{\text{H}}^{(0)}(t)\rangle = \text{Tr}\big[e^{iH^{X'}t/\hbar} X e^{-iH^{X'}t/\hbar}\rho(t_{\text{in}})\big] \tag{S28a}$$

$$\overset{t\to\infty}{=} 0 \tag{S28b}$$

which makes sense since any transient photonic amplitude will vanish due to photon escape. Similarly, at $O(H_{\text{int}})$, we have

$$\frac{\partial X_{\text{H}}^{(1)}(t)}{\partial t} - \frac{i}{\hbar}\big[H^{X'}, X_{\text{H}}^{(1)}(t)\big] = -\sqrt{\kappa_{\text{ex}}}\langle z_{\text{in,H}}^{a}(t)\rangle. \tag{S29}$$

This is a first-order inhomogeneous differential equation that can be solved with Green's function methods. We define

$$G^{XR}(t) = \Theta(t)\Big[X_{\text{H}}^{(0)}(t), X_{\text{H}}^{(0)\dagger}(0)\Big] \tag{S30}$$

which solves

$$\frac{\partial G^{XR}(t-t')}{\partial t} - \frac{i}{\hbar}\Big[H^{X'}, D^{X}(t-t')\Big] = \delta(t-t') \tag{S31}$$

where we need an additional assumption: the trace is performed over an initial state $\rho(t_{\text{in}})$ that is stationary with respect to $H^{X'}$; thus, it contains no photons. We readily obtain the Kubo linear response formula

$$\langle X^{(1)}(t)\rangle = -\sqrt{\kappa_{\text{ex}}}\int_{-\infty}^{\infty} dt_1 \langle z_{\text{in}}^{a}(t-t_1)\rangle D^{X}(t_1) \tag{S32a}$$

$$= -\sqrt{\kappa_{\text{ex}}}\int_{-\infty}^{\infty} dt_1 \langle z_{\text{in}}^{a}(t-t_1)\rangle \Theta(t_1)\langle\big[X_{\text{H}}^{(0)}(t_1), X^{\dagger}\big]\rangle, \tag{S32b}$$

where $D^{X}(t) = \langle G^{XR}(t)\rangle$ is the retarded Green's function. Importantly, Eq. S32b has the form of a convolution,

$$\langle X(\omega)\rangle = \langle X_{\text{H}}^{(1)}(\omega)\rangle = -i\sqrt{\kappa_{\text{ex}}}\langle z_{\text{in,H}}^{a}(\omega)\rangle D^{X}(\omega), \tag{S33}$$

where $D^X(\omega)$, according to the Fourier transform convention $f(\omega) = -i\int_{-\infty}^{\infty} dt e^{i\omega t} f(t)$, reads

$$D^X(\omega) = -i\int_{-\infty}^{\infty} dt e^{i\omega t}\Theta(t)\langle\left[e^{iH^{X'}t/\hbar} X e^{-iH^{X'}t/\hbar}, X^\dagger\right]\rangle. \tag{S34}$$

Incidentally, given the non-Hermitian nature of $H^{X'}$ due to photon leakage, $\rho(0)$ cannot contain photons, so one of the terms in the commutator is superfluous and the final propagator can be replaced, $H^{X'} \to H^X$,

$$D^X(\omega) = -i\int_{-\infty}^{\infty} dt e^{i\omega t}\Theta(t)\langle e^{iH^X t/\hbar} X e^{-iH^{X'}t/\hbar} X^\dagger\rangle. \tag{S35}$$

Similarly, for the anti-symmetric mode Y,

$$\langle Y(\omega)\rangle = -i\sqrt{\kappa_{\text{ex}}}\langle z^a_{\text{in,H}}(\omega)\rangle D^Y(\omega), \tag{S36a}$$

$$D^Y(\omega) = -i\int_{-\infty}^{\infty} dt e^{i\omega t}\Theta(t)\langle e^{iH^Y t/\hbar} Y e^{-iH^{Y'}t/\hbar} Y^\dagger\rangle. \tag{S36b}$$

Equation S35 and S36b can be fed into Eq. S33 and S36a. Using Eqs. S24,

$$T(\omega) = 1 + \kappa_{\text{ex}}\Im\left[D^X(\omega) + D^Y(\omega)\right] + \frac{\kappa_{\text{ex}}^2}{4}\left|D^X(\omega) + D^Y(\omega)\right|^2 \tag{S37a}$$

$$R(\omega) = \frac{\kappa_{\text{ex}}^2}{4}\left|D^X(\omega) - D^Y(\omega)\right|^2 \tag{S37b}$$

$$A(\omega) = \sum_{\alpha=X,Y} A^\alpha(\omega), \tag{S37c}$$

$$A^\alpha(\omega) = -\kappa_{\text{ex}}\Im\left[D^\alpha(\omega)\right] - \frac{\kappa_{\text{ex}}^2}{2}\left|D^\alpha(\omega)\right|^2. \tag{S37d}$$

We summarise the results of this section in Table S1.

3 Absorption spectra for arbitrary N molecules coupled to microtoroid resonator

According to Eq. S37d, there are two sources of absorption,

$$A^X(\omega) = -\kappa_{\text{ex}}\Im\left[D^X(\omega)\right] - \frac{\kappa_{\text{ex}}^2}{2}\left|D^X(\omega)\right|^2 \tag{S38a}$$

$$A^Y(\omega) = -\kappa_{\text{ex}}\Im\left[D^Y(\omega)\right] - \frac{\kappa_{\text{ex}}^2}{2}\left|D^Y(\omega)\right|^2 \tag{S38b}$$

Table S1: **Linear response of a microtoroidal resonator coupled to** N **molecules.** This table presents the expressions for transmission, reflection, and absorption in a microtoroidal resonator interacting with N molecules.

Response	Expression
Transmission	$T(\omega) = 1 + \kappa_{\text{ex}} \Im\left[D^X(\omega) + D^Y(\omega)\right] + \frac{\kappa_{\text{ex}}^2}{4}\left\|D^X(\omega) + D^Y(\omega)\right\|^2$
Reflection	$R(\omega) = \frac{\kappa_{\text{ex}}^2}{4}\left\|D^X(\omega) - D^Y(\omega)\right\|^2$
Absorption	$A(\omega) = -\kappa_{\text{ex}} \Im\left[D^X(\omega)\right] - \frac{\kappa_{\text{ex}}^2}{2}\left\|D^X(\omega)\right\|^2 - \kappa_{\text{ex}} \Im\left[D^Y(\omega)\right] - \frac{\kappa_{\text{ex}}^2}{2}\left\|D^Y(\omega)\right\|^2$

3.1 Absorption via anti-symmetric cavity mode

The absorption due to molecules is present only in $A^X(\omega)$ as only X couples with the molecules. On the other hand, $A^Y(\omega)$ accounts for the absorption due to the intrinsic loss in the microtoroidal cavity. Thus, it is straightforward to compute $D^Y(\omega)$ using Eq. S36b

$$D^Y(\omega) = -i \int_{-\infty}^{\infty} dt e^{i\omega t} \Theta(t) \langle e^{\frac{i}{\hbar} H^{Y'} t} Y e^{-\frac{i}{\hbar} H^{Y'} t} Y^\dagger \rangle \tag{S39a}$$

$$= \frac{1}{\omega - (\omega_{\text{ph}} - \beta) + i\left(\frac{\kappa_i + \kappa_{\text{ex}}}{2}\right)}. \tag{S39b}$$

Feeding Eq. S39b into Eq. S38b leads to,

$$A^Y(\omega) = \frac{\kappa_{\text{ex}} \kappa_i}{\left[\omega - (\omega_{\text{ph}} - \beta)\right]^2 + \left(\frac{\kappa_i + \kappa_{\text{ex}}}{2}\right)^2} \tag{S40}$$

3.2 Absorption via symmetric cavity mode

The absorption due to the symmetric mode X can be computed using

$$D^X(\omega) = -i \int_{-\infty}^{\infty} dt e^{i\omega t} \Theta(t) \langle e^{\frac{i}{\hbar} H^{X'} t} X e^{-\frac{i}{\hbar} H^{X'} t} X^\dagger \rangle, \tag{S41}$$

where $\langle \dots \rangle$ is taken with respect to the zero temperature photon-less initial state. Performing the Fourier transform, we obtain (*12*),

$$D^X(\omega) = \langle X G^X(\omega) X^\dagger \rangle, \tag{S42}$$

where $G^X(\omega) = \frac{1}{\omega - \tilde{H}^X + i0^+}$ is the total Green's function corresponding to the full Hamiltonian of the symmetric mode coupled to the N molecules.

4 Microresonator coupled to single molecule

The Green's function of the effective Hamiltonian for the symmetric optical mode of the microresonator in Eq. S21 is $G^X(\omega) = 1/(\omega - \tilde{H}^X + i0^+)$. Now, the Dyson equation,

$$G^X(\omega) = g(\omega) + g(\omega)VG^X(\omega). \tag{S43}$$

where, for single molecule, $g(\omega) = 1/(\omega - (\omega_{ph} + \beta) + i\frac{\kappa}{2})$ and $V = \tilde{g}X^\dagger|g\rangle\langle e| + h.c.$. Here, we considered $\tilde{g} = \sqrt{2}g$. Now, the Dyson series is

$$G^X(\omega) = g(\omega) + g(\omega)Vg(\omega) + g(\omega)Vg(\omega)Vg(\omega) + \dots \tag{S44}$$

As shown in Eq. S42, $\langle XG^X(\omega)X^\dagger\rangle$ is the total projection of the total Green's function onto the symmetric mode of the microtoroid. We will now compute the Dyson series term by term. Note that for this example, we have the microresonator off-resonant to the electronic transition of the molecule, $\Delta = |(\omega_{\text{ph}} + \beta) - \omega_e|$. Since the light-matter coupling $g \ll \Delta$, we are allowed to truncate the Dyson series (*31*).

We have the zeroth order term $\langle XG^X(\omega)X^\dagger\rangle = \langle Xg(\omega)X^\dagger\rangle = \frac{1}{\omega - (\omega_{\text{ph}} + \beta) + i\kappa/2}$ which is the Green's function of the bare cavity. For the higher-order terms of the Dyson expansion, we can use the technique of double-sided Feynman diagrams (DSFDs) (*12*). Note that the restriction to the linear response regime, conservation of excitation number by the Hamiltonian, and a photon-less zero temperature initial state only allows access to one of the sides of the diagram (we choose it to be the ket side by convention). Fig. S2 shows the DSFDs up to the fourth order term in V for a representative molecule with three levels: the ground electronic state with two vibrational levels $|g, \psi_0\rangle$ and $|g, \psi_1\rangle$, and the excited electronic state with one vibrational level $|e, \varphi_0\rangle$. We notice that the first and the third order terms in the series are zero. This is a known observation for subspace-projected Green's functions for coupled systems (*12, 32*). We obtain the expressions for the second

and fourth-order terms (where $D^X_{(n)}(\omega)$ represents the nth order term in the Dyson series) as

$$D^X_{(2)}(\omega) = \frac{1}{\omega - (\omega_{\text{ph}} + \beta) + i\kappa/2} g\langle\psi_0|\varphi_0\rangle \frac{1}{\omega - \omega_e + i\gamma/2} g\langle\varphi_0|\psi_0\rangle \frac{1}{\omega - (\omega_{\text{ph}} + \beta) + i\kappa/2}$$
$$= g^2\left[D^X_{(0)}(\omega)\right]^2 \chi^{(1)}(\omega), \tag{S45}$$

and,

$$D^X_{(4)}(\omega) = \frac{1}{\omega - (\omega_{\text{ph}} + \beta) + i\kappa/2} g\langle\psi_0|\varphi_0\rangle \frac{1}{\omega - \omega_e + i\gamma/2} g\langle\varphi_0|\psi_0\rangle \frac{1}{\omega - (\omega_{\text{ph}} + \beta) + i\kappa/2}$$
$$\times g\langle\psi_0|\varphi_0\rangle \frac{1}{\omega - \omega_e + i\gamma/2} g\langle\varphi_0|\psi_0\rangle \frac{1}{\omega - (\omega_{\text{ph}} + \beta) + i\kappa/2}$$
$$+ \frac{1}{\omega - (\omega_{\text{ph}} + \beta) + i\kappa/2} g\langle\psi_0|\varphi_0\rangle \frac{1}{\omega - \omega_e + i\gamma/2} g\langle\varphi_0|\psi_1\rangle \frac{1}{\omega - ((\omega_{\text{ph}} + \beta) + \omega_g) + i(\kappa + \gamma_{vib})/2}$$
$$\times g\langle\psi_1|\varphi_0\rangle \frac{1}{\omega - \omega_e + i\gamma/2} g\langle\varphi_0|\psi_0\rangle \frac{1}{\omega - (\omega_{\text{ph}} + \beta) + i\kappa/2}.$$
$$= g^4\left[D^X_{(0)}(\omega)\right]^3 \left[\chi^{(1)}(\omega)\right]^2 + R_{\text{vib}}(\omega) \tag{S46}$$

where, in general, $\chi^{(1)}(\omega) = -\lim_{\gamma\to 0^+} \sum_{n=0} \frac{|\langle\psi_0|\varphi_n\rangle|^2}{\omega - \omega_{e,n} + i\frac{\gamma}{2}}$ is the linear susceptibility of the molecule representing the cavity mediated Rayleigh-type scattering processes through which energy is dissipated into the molecular bath. And, the generalized form of $R_{\text{vib}}(\omega)$ is

$$R_{\text{vib}}(\omega) = g^4\left[D^X_{(0)}(\omega)\right]^2 \sum_{m=1}^{M_g} \frac{\langle\psi_0|\, G_{\text{ex}}(\omega)\, |\psi_m\rangle \langle\psi_m|\, G_{\text{ex}}(\omega)\, |\psi_0\rangle}{\omega - \left((\omega_{\text{ph}} + \beta) + \omega_{g,m}\right) + i\,\frac{(\kappa + \gamma_{\text{vib}})}{2}} \tag{S47}$$

Here, $G_{\text{ex}}(\omega) = \sum_m \frac{|\varphi_m\rangle\langle\varphi_m|}{(\omega - \omega_{e,m} + i\frac{\gamma}{2})}$ is the excited state Green's function of the molecule. Now, $R_{\text{vib}}(\omega)$ under standard approximations can be expressed in terms of Stokes and conditional anti-Stokes cross-section.

4.1 Stokes cross-section

Considering Real Franck-Condon Factors and $\gamma \to 0$,

$$R_{\text{vib}}(\omega) \approx g^4\left[D^X_{(0)}(\omega)\right]^2 \sum_{m=1}^{M_g} \frac{\left|\langle\psi_0|\, G_e(\omega)\, |\psi_m\rangle\right|^2}{\omega - ((\omega_{\text{ph}} + \beta) + \omega_{g,m}) + i\frac{(\kappa + \gamma_{\text{vib}})}{2}} \tag{S48}$$

Now, $R_{\text{vib}}(\omega)$ can be expressed in terms of Stokes Raman cross-section,

$$R_{\text{vib}}(\omega) \approx g^4\left[D^X_{(0)}(\omega)\right]^2 S_{\text{Raman}}(\omega_L = \omega, \omega_S = (\omega_{\text{ph}} + \beta)) \tag{S49}$$

where, $S_{\text{Raman}}(\omega_L = \omega, \omega_S = (\omega_{\text{ph}} + \beta))$ is the traditional Stokes Raman cross-section (*12, 18, 31*).

4.2 Conditional anti-Stokes cross-section

Considering Real Franck-Condon Factors and $\gamma \to 0$, $R_{\text{vib}}(\omega)$ around $(\omega_{\text{ph}} + \beta)$ is

$$R_{\text{vib}}(\omega) \approx g^4 \Big[D^X_{(0)}(\omega)\Big]^2 \sum_{m=1}^{M_g} \frac{\Big| \langle \psi_0 | \, G_e((\omega_{\text{ph}} + \beta)) \, |\psi_m\rangle \Big|^2}{\omega - ((\omega_{\text{ph}} + \beta) + \omega_{g,m}) + i\frac{(\kappa+\gamma_{\text{vib}})}{2}} \tag{S50}$$

Now, $R_{\text{vib}}(\omega)$ can be expressed in terms of Stokes Raman cross-section,

$$R_{\text{vib}}(\omega) \approx g^4 \Big[D^X_{(0)}(\omega)\Big]^2 S^C_{\text{Raman}}(\omega_L = (\omega_{\text{ph}} + \beta), \omega_{AS} = \omega) \tag{S51}$$

where, $S_{\text{Raman}}(\omega_L = \omega, \omega_S = (\omega_{\text{ph}} + \beta))$ is the conditional anti-Stokes Raman cross-section where the final state of the molecule is constrained to be in the global ground state (*12, 18, 31*).

4.3 Underlying Stokes and anti-Stokes mechanism

Thus, $R_{\text{vib}}(\omega)$ near the cavity peak can be expressed in terms of the underlying mechanism involving Stokes and subsequent anti-Stokes process

$$R_{\text{vib}}(\omega) \approx g^4 \Big[D^X_{(0)}(\omega)\Big]^2 \sqrt{S_{\text{Raman}}(\omega_L = \omega, \omega_S = (\omega_{\text{ph}} + \beta))} \sqrt{S^C_{\text{Raman}}(\omega_L = (\omega_{\text{ph}} + \beta), \omega_{AS} = \omega)} \tag{S52}$$

5 Exact solution for photon Green's function of a single molecule coupled to microresonator

It turns out that for the single molecule case, we can obtain an exact expression for the photon Green's function. For simplicity, we assume all the Franck-Condon factors to be 1 and write down all the higher-order terms in the Dyson expansion,

$$\frac{1}{\omega - (\omega_{\text{ph}} + \beta) + i\kappa/2} = A, \quad \frac{1}{\omega - \omega_e + i\gamma/2} = B, \quad \frac{1}{\omega - ((\omega_{\text{ph}} + \beta) + \omega_g) + i(\kappa + \gamma_{vib})/2} = C. \tag{S53}$$

Using a straightforward combinatorial counting of all the nonzero Feynman paths contributing to $\langle X G^X(\omega) X^\dagger \rangle$ at a given order, we obtain

$$D^X_{(0)}(\omega) = A, \tag{S54a}$$

$$D^X_{(2)}(\omega) = \left[AgBg\right]A, \tag{S54b}$$

$$D^X_{(4)}(\omega) = \left[AgBg\right]^2 A + Ag(BgCg)BgA, \tag{S54c}$$

$$D^X_{(6)}(\omega) = \left[AgBg\right]^3 A + Ag(BgCg)^2 BgA + AgBgCgBgAgBgA + AgBgAgBgCgBg, \tag{S54d}$$

and for arbitrary order,

$$D^X_{(2k)}(\omega) = \sum_{n=0}^{\infty} \sum_{m_n=0}^{\infty} \cdots \sum_{m_1=0}^{\infty} \left(Ag\left[(BgCg)^{m_1}\right]Bg\right)\left(Ag\left[(BgCg)^{m_2}\right]Bg\right)\ldots\left(Ag\left[(BgCg)^{m_n}\right]Bg\right)A \tag{S55}$$

such that $\sum_{i=1}^{n} m_i + n = k$ with $n \neq 0$. Summing over all the terms of this convergent series, we

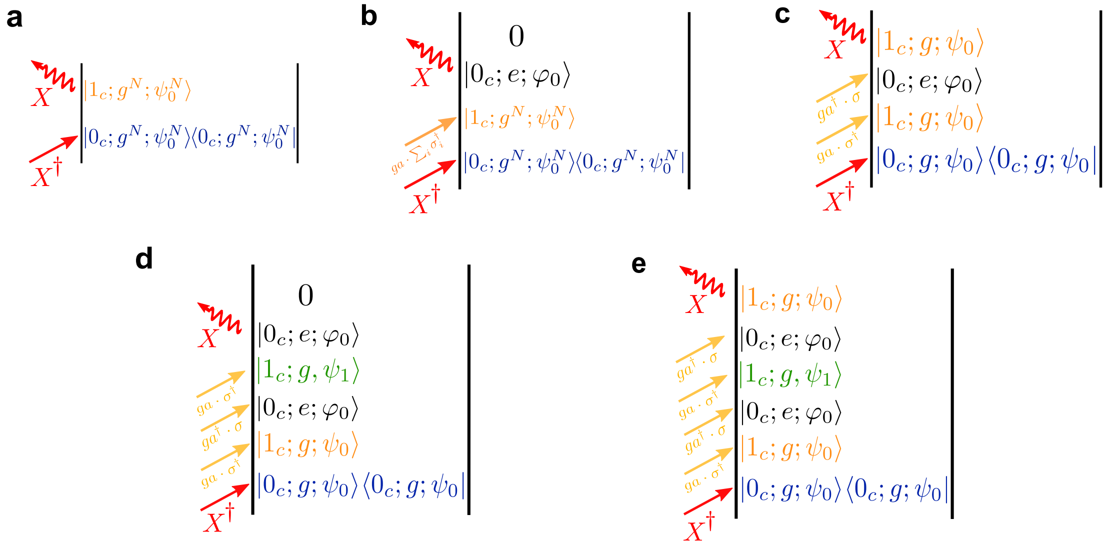

Figure S2: **Double-Sided Feynman diagrams for the single molecule coupled to the high-Q microresonator up to fourth order**: **a** Zeroth order, **b** first order, **c** second order, **d** third order, **e** fourth order terms in the light-matter couplings. Notice that the odd-order terms in the Dyson expansion vanish.

obtain

$$
\begin{aligned}
\langle XG^X(\omega)X^\dagger\rangle &= \sum_{n=0}^{\infty}\sum_{m_n=0}^{\infty}\cdots\sum_{m_1=0}^{\infty}\left(Ag\left[(BgCg)^{m_1}\right]Bg\right)\left(Ag\left[(BgCg)^{m_2}\right]Bg\right)\ldots\left(Ag\left[(BgCg)^{m_n}\right]Bg\right)A \\
&= \sum_{n=0}^{\infty}\left(Ag\left[\sum_{m=0}(BgCg)^m\right]Bg\right)^n A \\
&= \sum_{n=0}^{\infty}\left(Ag\frac{1}{1-BgCg}Bg\right)^n A = \left(\frac{1}{1-Ag\frac{1}{1-BgCg}Bg}\right)A. \qquad \text{(S56a)}
\end{aligned}
$$

6 Dyson expansion for N molecules coupled to the microresonator

Let the zero temperature initial state be

$$
|\Psi_0\rangle = |0_c; g^N; \psi_0^N\rangle, \tag{S57}
$$

where $|g^N\rangle = |ggg\ldots g\rangle$ and $|\psi_0^N\rangle = |\psi_0\psi_0\ldots\psi_0\rangle$ represents all the N molecules in $|g,\psi_0\rangle$ state. Similar to the previous case, we can compute the terms of the Dyson series. Further, we also have the odd order terms in the Dyson expansion to be zero. Now, we write the terms of the series order-by-order using the DSFDs in Fig. S3. We have,

$$D^{X(N)}_{(2)}(\omega) = \frac{1}{\omega - (\omega_{\text{ph}} + \beta) + i\kappa/2} g\sqrt{N}\langle\psi_0|\varphi_0\rangle \frac{1}{\omega - \omega_e + i\gamma/2} g\sqrt{N}\langle\varphi_0|\psi_0\rangle \frac{1}{\omega - (\omega_{\text{ph}} + \beta) + i\kappa/2}, \tag{S58}$$

$$\begin{aligned} D^{X(N)}_{(4)}(\omega) = & \frac{1}{\omega - (\omega_{\text{ph}} + \beta) + i\kappa/2} \left(g\sqrt{N}\langle\psi_0|\varphi_0\rangle \frac{1}{\omega - \omega_e + i\gamma/2} g\sqrt{N}\langle\varphi_0|\psi_0\rangle \frac{1}{\omega - (\omega_{\text{ph}} + \beta) + i\kappa/2} \right)^2 \\ & + \frac{1}{\omega - (\omega_{\text{ph}} + \beta) + i\kappa/2} g\sqrt{N}\langle\psi_0|\varphi_0\rangle \frac{1}{\omega - \omega_e + i\gamma/2} g\langle\varphi_0|\psi_1\rangle \\ & \times \frac{1}{\omega - ((\omega_{\text{ph}} + \beta) + \omega_g) + i(\kappa + \gamma_{vib})/2} g\langle\psi_1|\varphi_0\rangle \frac{1}{\omega - \omega_e + i\gamma/2} \\ & \times g\sqrt{N}\langle\varphi_0|\psi_0\rangle \frac{1}{\omega - (\omega_{\text{ph}} + \beta) + i\kappa/2}, \end{aligned} \tag{S59}$$

$$\begin{aligned} D^{X(N)}_{(6)}(\omega) = & \frac{1}{\omega - (\omega_{\text{ph}} + \beta) + i\kappa/2} \left(g\sqrt{N}\langle\psi_0|\varphi_0\rangle \frac{1}{\omega - \omega_e + i\gamma/2} g\sqrt{N}\langle\varphi_0|\psi_0\rangle \frac{1}{\omega - (\omega_{\text{ph}} + \beta) + i\kappa/2} \right)^3 + \\ & + \frac{1}{\omega - (\omega_{\text{ph}} + \beta) + i\kappa/2} g\sqrt{N}\langle\psi_0|\varphi_0\rangle \left(\frac{1}{\omega - \omega_e + i\gamma/2} g\langle\varphi_0|\psi_1\rangle \right. \\ & \left. \frac{1}{\omega - ((\omega_{\text{ph}} + \beta) + \omega_g) + i(\kappa + \gamma_{vib})/2} g\langle\psi_1|\varphi_0\rangle \right)^2 \frac{1}{\omega - \omega_e + i\gamma/2} \\ & \times g\sqrt{N}\langle\varphi_0|\psi_0\rangle \frac{1}{\omega - (\omega_{\text{ph}} + \beta) + i\kappa/2} \\ & + \frac{1}{\omega - (\omega_{\text{ph}} + \beta) + i\kappa/2} g\sqrt{N}\langle\psi_0|\varphi_0\rangle \frac{1}{\omega - \omega_e + i\gamma/2} g\langle\varphi_0|\psi_1\rangle \\ & \times \frac{1}{\omega - ((\omega_{\text{ph}} + \beta) + \omega_g) + i(\kappa + \gamma_{vib})/2} \\ & \times \left(g\sqrt{N-1}\langle\psi_0|\varphi_0\rangle \frac{1}{\omega - (\omega_e + \omega_g) + i(\gamma + \gamma_{vib})/2} \right. \\ & \left. \times g\sqrt{N-1}\langle\varphi_0|\psi_0\rangle \frac{1}{\omega - ((\omega_{\text{ph}} + \beta) + \omega_g) + i(\kappa + \gamma_{vib})/2} \right) \\ & \times g\langle\psi_1|\varphi_0\rangle \frac{1}{\omega - \omega_e + i\gamma/2} g\sqrt{N}\langle\varphi_0|\psi_0\rangle \frac{1}{\omega - (\omega_{\text{ph}} + \beta) + i\kappa/2} \end{aligned} \tag{S60}$$

Now assuming N is large, such that $g\sqrt{N-1} \approx g\sqrt{N}$, we see that there are two emergent timescales in the problem: the collective timescale, $g\sqrt{N}$, that involves only the linear susceptibility of the molecular ensemble, and the single molecule timescale, g, that mediates the Raman processes. Since $g\sqrt{N} \gg g$, and from our experience with the single molecule case, we expect the Raman terms to be proportional to g^4 (which is essentially $g^2N \times g^2$). Thus, the term in the Dyson expansion that accounts for $2k$ orders in collective timescale and 2 orders in single molecule timescale (this

can also be checked in a similar way using a combinatorial counting of Feynman paths Fig. S3,

$$
\begin{aligned}
D^{X(N)}_{(2k)}(\omega) &= \frac{1}{\omega-(\omega_{\text{ph}}+\beta)+i\kappa/2}\left(g\sqrt{N}\langle\psi_0|\varphi_0\rangle\frac{1}{\omega-\omega_e+i\gamma/2}g\sqrt{N}\langle\varphi_0|\psi_0\rangle\frac{1}{\omega-(\omega_{\text{ph}}+\beta)+i\kappa/2}\right)^k \\
&+\sum_{n=0}^{k}\sum_{m_2=0}^{k}\sum_{m_1=0}^{k}\frac{1}{\omega-(\omega_{\text{ph}}+\beta)+i\kappa/2}\left(g\sqrt{N}\langle\psi_0|\varphi_0\rangle\frac{1}{\omega-\omega_e+i\gamma/2}g\sqrt{N}\langle\varphi_0|\psi_0\rangle\frac{1}{\omega-(\omega_{\text{ph}}+\beta)+i\kappa/2}\right)^{m_1} \\
&\times\Bigg[g\sqrt{N}\langle\psi_0|\varphi_0\rangle\frac{1}{\omega-\omega_e+i\gamma/2}g\langle\varphi_0|\psi_1\rangle\frac{1}{\omega-((\omega_{\text{ph}}+\beta)+\omega_g)+i(\kappa+\gamma_{vib})/2} \\
&\times\left(g\sqrt{N}\langle\psi_0|\varphi_0\rangle\frac{1}{\omega-(\omega_e+\omega_g)+i(\gamma+\gamma_{vib})/2}g\sqrt{N}\langle\varphi_0|\psi_0\rangle\frac{1}{\omega-((\omega_{\text{ph}}+\beta)+\omega_g)+i(\kappa+\gamma_{vib})/2}\right)^n \\
&\times g\langle\psi_1|\varphi_0\rangle\frac{1}{\omega-\omega_e+i\gamma/2}g\sqrt{N}\langle\varphi_0|\psi_0\rangle\Bigg]\frac{1}{\omega-(\omega_{\text{ph}}+\beta)+i\kappa/2} \\
&\times\left(g\sqrt{N}\langle\psi_0|\varphi_0\rangle\frac{1}{\omega-\omega_e+i\gamma/2}g\sqrt{N}\langle\varphi_0|\psi_0\rangle\frac{1}{\omega-(\omega_{\text{ph}}+\beta)+i\kappa/2}\right)^{m_2}+O(g^4),
\end{aligned}
\tag{S61}
$$

such that $m_1+m_2+n+2=k$. Here, the reader can verify that the higher order terms in g, *e.g.* $O(g^6)$ for the case of N molecules coupled to the resonator contain new vacuum-mediated features that are not present in the single-molecule case. Since g is small, these features are even smaller than the sought-out Raman peaks. Thus, observing these peaks requires ultrahigh Q cavities, which are a topic for future work.

Further, the key difference between the case of N molecules and a single molecule coupled to the resonator is the emergence of a collective fast timescale that forbids truncation of terms involving powers of $g\sqrt{N}$ (as $g\sqrt{N}$ leads to a divergent series). Thus, this necessitates the exact resummation of these collective terms. The issue of such apparent divergences is well known in the literature of Quantum electrodyanmics (QED) and Quantum chromodyanmics (QCD) where several asymptotic techniques have been developed to do exact resummation. We use the method of Borel summation shown in the Appendix to resum the series. We obtain,

$$
\begin{aligned}
D^{X(N)}_{(0)}(\omega) = S_1 &= \sum_{k=0}^{\infty}\frac{1}{\omega-(\omega_{\text{ph}}+\beta)+i\kappa/2}\left(g\sqrt{N}\langle\psi_0|\varphi_0\rangle\frac{1}{\omega-\omega_e+i\gamma/2}g\sqrt{N}\langle\varphi_0|\psi_0\rangle\frac{1}{\omega-(\omega_{\text{ph}}+\beta)+i\kappa/2}\right)^k, \\
&= \frac{1}{\omega-(\omega_{\text{ph}}+\beta)+i\kappa/2-g^2N\frac{|\langle\psi_0|\varphi_0\rangle|^2}{\omega-\omega_e+i\gamma/2}}
\end{aligned}
\tag{S62}
$$

Similarly, using Borel summation,

$$D^{X(N)}_{(2)}(\omega) = S_2 = D^{X(N)}_{(0)}(\omega)\Big[g\sqrt{N}\langle\psi_0|\varphi_0\rangle\frac{1}{\omega-\omega_e+i\gamma/2}g\langle\varphi_0|\psi_1\rangle$$

$$\times\frac{1}{\omega-((\omega_{\text{ph}}+\beta)+\omega_g)+i(\kappa+\gamma_{vib})/2}g\sqrt{N}\langle\psi_0|\varphi_0\rangle \tag{S63}$$

$$\times\frac{1}{\omega-((\omega_{\text{ph}}+\beta)+\omega_{vib})+i(\kappa+\gamma_{vib})/2-g^2N\frac{|\langle\psi_0|\varphi_0\rangle|^2}{\omega-(\omega_e+\omega_{vib})+i(\gamma+\gamma_{vib})/2}}g\langle\psi_1|\varphi_0\rangle \tag{S64}$$

$$\times\frac{1}{\omega-\omega_e+i\gamma/2}g\sqrt{N}\langle\varphi_0|\psi_0\rangle\Big]D^{X(N)}_{(0)}(\omega). \tag{S65}$$

This is the final expression we obtain for the photon Green's function up to g^2. We have,

$$D^{X(N)}(\omega) = D^{X(N)}_{(0)}(\omega) + D^{X(N)}_{(2)}(\omega) + O(g^4). \tag{S66}$$

Here,

$$D^{X(N)}_{(2)}(\omega) = (g\sqrt{N})^2g^2\Big[D^{X(N)}_{(0)}(\omega)\Big]^2\sum_{m=1}^{M_g}\frac{\langle\psi_0|\,G_{\text{ex}}(\omega)\,|\psi_m\rangle\,\langle\psi_m|\,G_{\text{ex}}(\omega)\,|\psi_0\rangle}{\omega-((\omega_{\text{ph}}+\beta)+\omega_{g,m})+i\frac{(\kappa+\gamma_{\text{vib}})}{2}-g^2N\frac{|\langle\psi_0|\varphi_0\rangle|^2}{\omega-(\omega_e+\omega_{g,m})+i\frac{(\gamma+\gamma_{vib})}{2}}} \tag{S67}$$

6.1 Stokes cross-section

Considering real Franck-Condon factors and $\gamma\longrightarrow 0$, we write $D^{X(N)}_{(2)}(\omega)$ near $\omega=\Omega$, where Ω is the lowest peak in the absorption spectra,

$$D^{X(N)}_{(2)}(\omega) \approx (g\sqrt{N})^2g^2\Big[\frac{1}{\omega-\Omega+i\frac{\Gamma}{2}}\Big]^2\sum_{m=1}^{M_g}\frac{\Big|\langle\psi_0|\,G_{\text{ex}}(\omega)\,|\psi_m\rangle\Big|^2}{\omega-(\Omega+\omega_{g,y})+i\frac{\Gamma}{2}} \tag{S68a}$$

$$\approx (g\sqrt{N})^2g^2\Big[D^{X(N)}_{(0)}(\omega)\Big]^2 S_{\text{Raman}}(\omega_L=\omega,\omega_S=\Omega) \tag{S68b}$$

6.2 Conditional anti-Stokes cross-section

Considering real Franck-Condon factors and $\gamma\longrightarrow 0$,we write $D^{X(N)}_{(2)}(\omega)$ near $\omega=\Omega$, where Ω is the lowest peak in the absorption spectra,

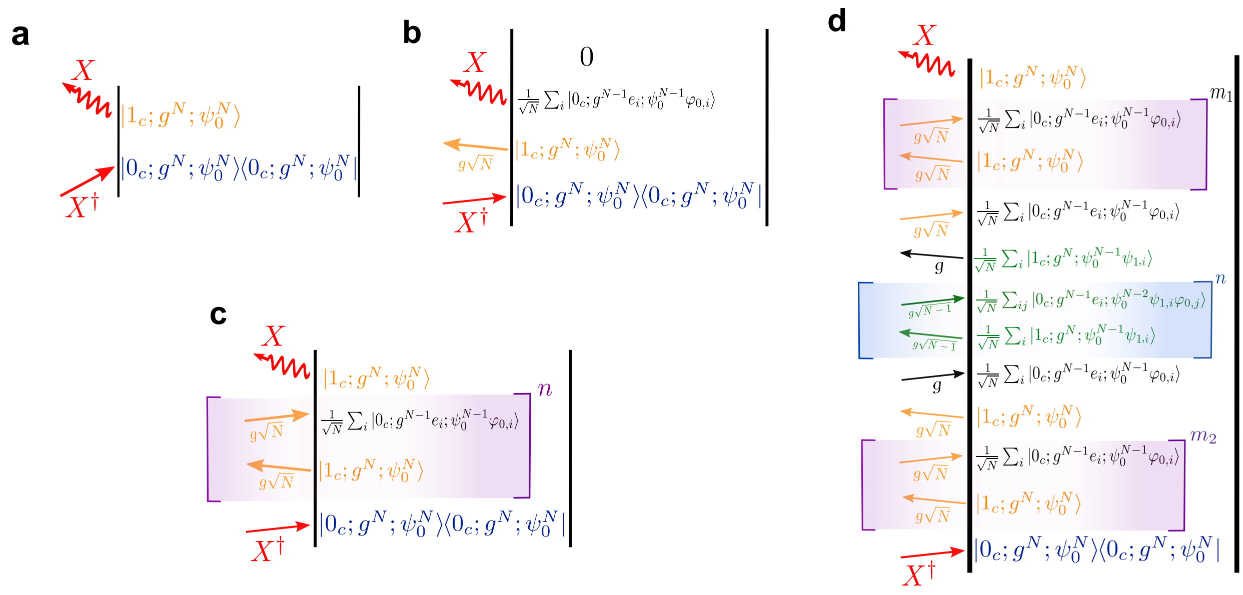

Figure S3: **Double-Sided Feynman diagrams for the N molecule coupled to the high-Q microresonator up to second order in g**: **a** Zeroth order, **b** first order, (c) second order, (d) third order, (e) fourth order terms in the light-matter couplings. Notice that the odd-order terms in the Dyson expansion vanish.

$$D_{(2)}^{X(N)}(\omega) \approx (g\sqrt{N})^2 g^2 \Big[\frac{1}{\omega - \Omega + i\frac{\Gamma}{2}}\Big]^2 \sum_{m=1}^{M_g} \frac{\Big|\langle\psi_0| G_{\text{ex}}(\Omega)\, |\psi_m\rangle\Big|^2}{\omega - (\Omega + \omega_{g,y}) + i\frac{\Gamma}{2}} \tag{S69a}$$

$$\approx (g\sqrt{N})^2 g^2 \Big[D_{(0)}^{X(N)}(\omega)\Big]^2 S_{\text{Raman}}^{C}(\omega_L = \Omega, \omega_{AS} = \omega) \tag{S69b}$$

6.3 Underlying Stokes and anti-Stokes mechanism

Thus, $D_{(2)}^{X(N)}(\omega)$ near the lower absorption peak (Ω) can be expressed in terms of the underlying mechanism involving Stokes and subsequent anti-Stokes process

$$D_{(2)}^{X(N)}(\omega) \approx (g\sqrt{N})^2 g^2 \Big[D_{(0)}^{X(N)}(\omega)\Big]^2 \sqrt{S_{\text{Raman}}(\omega_L = \omega, \omega_S = \Omega)}\sqrt{S_{\text{Raman}}^{C}(\omega_L = \Omega, \omega_{AS} = \omega)} \tag{S70}$$

Table S2: **Vibrational mode parameters of isoprene (*33*).** The Raman spectrum of isoprene is modeled using ten harmonic oscillators, whose frequencies (ω), displacements (Δ), and tentative chemical assignments (*81*) are summarized below.

ω (eV)	Δ	Assignment
0.202	1.77	C=C stretching
0.198	-0.39	C=C stretching
0.181	0.36	CH_3 asymmetric deformation; CH_2 scissoring
0.180	-0.30	CH_3 asymmetric deformation
0.177	-0.34	CH_2 scissoring; CH bend
0.172	-0.35	CH_2 scissoring
0.161	-1.01	CH bend
0.131	0.41	CH_2 rocking; C−C=C bend; CH bend
0.123	0.37	CH bend; CH_2 wagging; CH_2 twisting
0.117	0.37	CH_2 twisting; CH_2 wagging

7 Calculation of Light-Matter Coupling Strength (g)

The single-molecule light-matter coupling strength, g, is given by (*36*)

$$g = |d_{12}|\sqrt{\frac{(\omega_{\mathrm{ph}} + \beta)}{2\hbar\epsilon_0 V_m}} \tag{S71}$$

where $|d_{12}|$ is the electronic transition dipole moment of the molecule, $(\omega_{\mathrm{ph}} + \beta)$ is the cavity frequency, ϵ_0 is the permittivity of free space, and V_m is the cavity mode volume.

For isoprene, the transition dipole moment is calculated using its characteristic transition wavelength, $\lambda_{12} = 205.74$ nm, and oscillator strength, $f_{12} = 0.5523$ (*37*),

$$f_{12} = \frac{2}{3}\frac{m_e}{\hbar^2}E_{21}|r_{21}|^2, \tag{S72}$$

, which gives

$$|d_{12}| = 1e \times |r_{21}| \tag{S73a}$$

$$= 1e \times \sqrt{\frac{3\hbar^2 f_{12}\lambda_{12}}{2m_e hc}} \tag{S73b}$$

$$= 5\mathrm{D}. \tag{S73c}$$

The cavity is red-detuned by 0.3 eV relative to the electronic transition of isoprene, yielding

$$(\omega_{\mathrm{ph}} + \beta) = \omega_{eg} - 0.3\,\mathrm{eV} = 5.726\,\mathrm{eV}. \tag{S74}$$

The mode volume is taken as six times the diffraction limit for a microtoroidal cavity with refractive index $n = 1.44$:

$$V_{\mathrm{m}} = 6 \times V_{\mathrm{diff}} = 6 \times \left(\frac{\lambda}{n}\right)^3 = 21.2 \times 10^{-3}\,\mu\mathrm{m}^3. \tag{S75}$$

Substituting these values, the light-matter coupling strength is

$$g = |d_{12}|\sqrt{\frac{(\omega_{\mathrm{ph}} + \beta)}{2\hbar\epsilon_0 V_m}} \tag{S76a}$$

$$= 1.6 \times 10^{-4}\,\mathrm{eV}. \tag{S76b}$$

8 Maximum Number of Molecules coupled to a microtoroidal resonator

The maximum number of molecules coupled to the resonator is computed using the surface density of the molecules. The surface density of monolayer of receptors is $\rho = 5 \times 10^{14}$ (*46*). Thus, the maximum number of molecules coupled to the resonator is

$$\begin{aligned} N_{\max} &= \rho \times A && \text{(S77a)} \\ &= \rho \times \pi^2 \times D_{\lambda_c} \times d_{\lambda_c} && \text{(S77b)} \\ &= 5 \times 10^{14} \times 10^4 \times \pi^2 \times 10^{-6} \times 0.2 \times 10^{-6} && \text{(S77c)} \\ &\approx 10^7 \text{ molecules} && \text{(S77d)} \end{aligned}$$

9 Appendix: Borel summation

Here, we restate the mathematics behind Borel summation for completeness (*47,48*). Let us consider the following series:

$$S = A \sum_{n=0}^{\infty} B^n. \tag{S78}$$

We define a slightly different power series in an auxiliary variable λ:

$$f(\lambda) = A \sum_{n=0}^{\infty} (B\lambda)^n, \tag{S79}$$

where setting $\lambda = 1$ recovers the original sum. The Borel transform $\hat{f}(t)$ is defined by replacing each coefficient $a_n = B^n A$ with $\frac{a_n}{n!} t^n$. Conversely:

$$\hat{f}(t) = \sum_{n=0}^{\infty} \frac{a_n}{n!} t^n = \sum_{n=0}^{\infty} \frac{B^n A}{n!} t^n = A \sum_{n=0}^{\infty} \frac{B^n}{n!} t^n = A e^{Bt}. \tag{S80}$$

The Borel sum $\mathcal{B}[f(\lambda)]$ of $f(\lambda)$ is then the Laplace-type integral

$$\mathcal{B}[f(\lambda)] = \int_0^{\infty} e^{-u} \hat{f}(\lambda u) du. \tag{S81}$$

In our case,

$$\hat{f}(\lambda u) = Ae^{B(\lambda u)}, \tag{S82}$$

so

$$\begin{aligned}\mathcal{B}[f(\lambda)] &= \int_0^{\infty} e^{-u} A e^{B(\lambda u)} du, \\ &= A \int_0^{\infty} e^{u(B\lambda - 1)} du.\end{aligned}$$

The original sum S corresponds to $\lambda = 1$, so we set $\lambda = 1$:

$$S_{\text{Borel}} = A \int_0^{\infty} e^{u(B-1)} du. \tag{S83}$$

Now if $\Re(B) < 1$ the exponential $e^{u(B-1)}$ decays for $u > 0$, so the integral converges and equals $\frac{A}{1-B}$. If $\Re(B) \geq 1$: then $\Re(B-1) \geq 0$ and the real-axis integral $\int_0^{\infty} e^{u(B-1)} du$ diverges. However, there are complex analysis tricks to analytically continue to the space of $\Re(B) < 1$. Rather than integrating along the positive real axis $t \in [0, \infty)$, one rotates the integration contour into the complex plane in such a way that the real part of the exponent becomes negative, ensuring convergence.

Let $M = B - 1$. If $\Re(M) > 0$, the real-axis integral $\int_0^{\infty} e^{Mt} dt$ diverges. However, consider integrating along the line $t = re^{i\theta}$ for r from 0 to ∞, where θ is chosen so that,

$$\Re[M(re^{i\theta})] = r\Re[Me^{i\theta}] \quad < 0, \tag{S84}$$

ensuring the exponential decays at large r. Concretely, if M is positive real number (*i.e.* $B > 1$ real), you can take $\theta = \pi$ (which amounts to integration along the negative real axis). Then

$$t = re^{i\pi} = -r, \quad r \in [0, \infty), \tag{S85}$$

so $\Re(M \cdot (-r)) = -r\Re(M) < 0$. That yields convergence. Along the line $t = re^{i\theta}$, we have $dt = e^{i\theta}dr$. So formally:

$$\int_0^\infty e^{(B-1)t}dt \to \int_0^\infty e^{(B-1)re^{i\theta}}e^{i\theta}dr. \tag{S86}$$

If we choose θ so that $\Re((B-1)e^{i\theta}) < 0$ (e.g. $\theta = \pi$ when $B > 1$ and is purely real), the integral converges. We then have,

$$\int_0^\infty e^{(B-1)t}dt = \int_C e^{(B-1)t}dt, \tag{S87}$$

provided there are no singularities or branch cuts crossing the path. In fact, it turns out that the integral value is the same as if we had $\Re(B) < 1$. One recovers,

$$S_{\text{Borel}} = A\int_{\mathbb{C}} e^{(B-1)t}dt = \frac{A}{1-B}, \tag{S88}$$

which is the same answer as one would get without the analytic continuation.

REFERENCES

1. M. B. James, D. J. Griffiths, Why the speed of light is reduced in a transparent medium. *Am. J. Phys.* **60**, 309–313 (1992).

2. J. D. Jackson, *Classical Electrodynamics* (Wiley, Hoboken, NJ, ed. 3, 1998).

3. B. E. A. Saleh, M. C. Teich, *Fundamentals of Photonics* (Wiley, New York, NY, ed. 2, 1991).

4. A. Yariv, P. Yeh, *Photonics: Optical Electronics in Modern Communications* (Oxford University Press, New York, NY, ed. 6, 2006).

5. N. Német, D. White, S. Kato, S. Parkins, T. Aoki, Transfer-matrix approach to determining the linear response of all-fiber networks of cavity-QED systems. *Phys. Rev. Appl.* **13**, 064010 (2020).

6. R. J. Glauber, M. Lewenstein, Quantum optics of dielectric media. *Phys. Rev. A* **43**, 467–491 (1991).

7. D. Gatto Monticone, K. Katamadze, P. Traina, E. Moreva, J. Forneris, I. Ruo-Berchera, P. Olivero, I. P. Degiovanni, G. Brida, M. Genovese, Beating the Abbe diffraction limit in confocal microscopy via nonclassical photon statistics. *Phys. Rev. Lett.* **113**, 143602 (2014).

8. F. M. Kalarde, F. Ciccarello, C. S. Muñoz, J. Feist, C. Galland, Photon antibunching in single-molecule vibrational sum-frequency generation. *Nanophotonics* **14**, 59–73 (2025).

9. M. D. Anderson, S. Tarrago Velez, K. Seibold, H. Flayac, V. Savona, N. Sangouard, C. Galland, Two-color pump-probe measurement of photonic quantum correlations mediated by a single phonon. *Phys. Rev. Lett.* **120**, 233601 (2018).

10. K. J. Vahala, Optical microcavities. *Nature* **424**, 839–846 (2003).

11. P. Brumer, M. Shapiro, One photon mode selective control of reactions by rapid or shaped laser pulses: An emperor without clothes? *Chem. Phys.* **139**, 221–228 (1989).

12. S. Mukamel, *Principles of Nonlinear Optical Spectroscopy* (Oxford University Press, 1995).

13. T. Yoshie, L. Tang, S.-Y. Su, Optical microcavity: Sensing down to single molecules and atoms. *Sensors* **11**, 1972–1991 (2011).

14. M. Zhou, S. M. Shahriar, Optomechanical resonator as a negative dispersion medium for enhancing the sensitivity bandwidth in a gravitational-wave detector. *Phys. Rev. D* **98**, 022003 (2018).

15. L. A. Kuznetsova, W. T. Coakley, Microparticle concentration in short path length ultrasonic resonators: Roles of radiation pressure and acoustic streaming. *J. Acoust. Soc. Am.* **116**, 1956–1966 (2004).

16. D. Rho, C. Breaux, S. Kim, Label-free optical resonator-based biosensors. *Sensors* **20**, 5901 (2020).

17. G. Schatz, M. Ratner, *Quantum Mechanics in Chemistry* (Dover Books on ChemistryDover Publications, 2002).

18. B. Dayan, A. S. Parkins, T. Aoki, E. P. Ostby, K. J. Vahala, H. J. Kimble, A photon turnstile dynamically regulated by one atom. *Science* **319**, 1062–1065 (2008).

19. E. M. Purcell, Spontaneous emission probabilities at radio frequencies. *Phys. Rev.* **69**, 681 (1946).

20. B. Petrak, N. Djeu, A. Muller, Purcell-enhanced Raman scattering from atmospheric gases in a high-finesse microcavity. *Phys. Rev. A* **89**, 023811 (2014).

21. C. W. Gardiner, M. J. Collett, Input and output in damped quantum systems: Quantum stochastic differential equations and the master equation. *Phys. Rev. A* **31**, 3761–3774 (1985).

22. C. Ciuti, I. Carusotto, Input-output theory of cavities in the ultrastrong coupling regime: The case of time-independent cavity parameters. *Phys. Rev. A* **74**, 033811 (2006).

23. D. Steck, *Quantum and Atom Optics* (University of Oregon, 2007).

24. H. Li, A. Piryatinski, J. Jerke, A. R. S. Kandada, C. Silva, E. R. Bittner, Probing dynamical symmetry breaking using quantum-entangled photons. *Quantum Sci. Technol.* **3**, 015003 (2018).

25. J. Yuen-Zhou, A. Koner, Linear response of molecular polaritons. *J. Chem. Phys.* **160**, 154107 (2024).

26. D. K. Armani, T. J. Kippenberg, S. M. Spillane, K. J. Vahala, Ultra-high-Q toroid microcavity on a chip. *Nature* **421**, 925–928 (2003).

27. J. A. Ćwik, P. Kirton, S. De Liberato, J. Keeling, Excitonic spectral features in strongly coupled organic polaritons. *Phys. Rev. A* **93**, 033840 (2016).

28. M. A. Zeb, P. G. Kirton, J. Keeling, Exact states and spectra of vibrationally dressed polaritons. *ACS Photonics* **5**, 249–257 (2018).

29. J. Richter, "Ultra-high-Q inverted silica microtoroid resonators monolithically integrated into a silicon photonics platform," thesis, Rheinisch-Westfälische Technische Hochschule Aachen (2018).

30. D. Tannor, *Introduction to Quantum Mechanics: A Time Dependent Perspective* (University Science Books, Melville, 2007).

31. F.-M. Dittes, The decay of quantum systems with a small number of open channels. *Phys. Rep.* **339**, 215–316 (2000).

32. D. J. Tannor, E. J. Heller, Polyatomic Raman scattering for general harmonic potentials. *J. Chem. Phys.* **77**, 202–218 (1982).

33. A. B. Myers, R. A. Mathies, D. J. Tannor, E. J. Heller, Excited state geometry changes from preresonance Raman intensities: Isoprene and hexatriene. *J. Chem. Phys.* **77**, 3857–3866 (1982).

34. S. Gao, S. Wang, C. Gu, J. Zhu, R. Zhang, Y. Guo, Y. Yan, B. Zhou, Study on the measurement of isoprene by differential optical absorption spectroscopy. *Atmos. Meas. Tech.* **14**, 2649–2657 (2021).

35. J. Heebner, R. Grover, T. Ibrahim, "Optical microresonators: Theory, fabrication, and applications" in *Optical Sciences* (Springer, 2008), vol. 138.

36. G. Martins, A. M. Ferreira-Rodrigues, F. N. Rodrigues, G. G. B. de Souza, N. J. Mason, S. Eden, D. Duflot, J. P. Flament, S. V. Hoffmann, J. Delwiche, M. J. Hubin-Franskin, P. Limão-Vieira, Valence shell electronic spectroscopy of isoprene studied by theoretical calculations and by electron scattering, photoelectron, and absolute photoabsorption measurements. *Phys. Chem. Chem. Phys.* **11**, 11219–11231 (2009).

37. E. P. Ostby, "Photonic whispering-gallery resonators in new environments," thesis, California Institute of Technology (2009).

38. A. W. Chin, A. Rivas, S. F. Huelga, M. B. Plenio, Exact mapping between system-reservoir quantum models and semi-infinite discrete chains using orthogonal polynomials. *J. Math. Phys.* **51**, 092109 (2010).

39. D. Long, *The Raman Effect: A Unified Treatment of the Theory of Raman Scattering by Molecules* (Wiley, 2002).

40. R. Paschotta, "Shot noise," RP Photonics Encyclopedia (2025), www.rp-photonics.com/shot_noise.html.

41. Thorlabs, "Photodiodes" (2023), www.thorlabs.com/photodiodes.

42. Hamamatsu Photonics, "Si photodiodes" (2025), www.hamamatsu.com/content/dam/hamamatsu-photonics/sites/documents/99_SALES_LIBRARY/ssd/si_pd_kspd9001e.pdf.

43. T. J. Fellers, M. W. Davidson, "CCD saturation and blooming" (2016), https://hamamatsu.magnet.fsu.edu/articles/ccdsatandblooming.html.

44. J. Clark, “The Beer-Lambert law” (LibreTexts Chemistry, 2023), https://chem.libretexts.org/Bookshelves/Physical_and_Theoretical_Chemistry_Textbook_Maps/Supplemental_Modules_(Physical_and_Theoretical_Chemistry)/Spectroscopy/Electronic_Spectroscopy/Electronic_Spectroscopy_Basics/The_Beer-Lambert_Law.

45. L. Zhuravlev, Concentration of hydroxyl groups on the surface of amorphous silicas. *Langmuir* **3**, 316–318 (1987).

46. G. H. Hardy, *Divergent Series* (American Mathematical Society, 2024), vol. 334.

47. E. Borel, “Mémoire sur les séries divergentes” in *Annales scientifiques de l'École Normale Supérieure*. (1899), vol. 16, pp. 9–131.

48. L. Ciura, A. Kolek, W. Gawron, A. Kowalewski, D. Stanaszek, Measurements of low frequency noise of infrared photo-detectors with transimpedance detection system. *Metrol. Meas. Syst.* **21**, 461–472 (2014).

49. R. Kiely, *Understanding and Eliminating 1/f Noise* (Analog Dialogue, 2019).

50. A. Descloux, K. S. Grußmayer, A. Radenovic, 3D active stabilization for single-molecule imaging. *Nat. Protoc.* **16**, 256–308 (2021).

51. Zurich Instruments, “Principles of lock-in detection for photodiode signals” (Zurich Instruments, 2025), https://cms.zhinst.com/sites/default/files/documents/2025-10/zi_whitepaper_principles_of_lock-in_detection.pdf.

52. E. Robinson, J. Trägårdh, I. Lindsay, H. Gersen, Balanced detection for interferometry with a noisy source. *Rev. Sci. Instrum.* **83**, 063705 (2012).

53. Herda Radio, “Demystifying demodulation: Techniques unveiled” (2023), https://herdaradio.com/no/blog/radioknowledge/demodulation-techniques-unveiled/.

54. M. Jin, S. J. Tang, J. H. Chen, X. C. Yu, H. Shu, Y. Tao, A. K. Chen, Q. Gong, X. Wang, Y. F. Xiao, 1/f-noise-free optical sensing with an integrated heterodyne interferometer. *Nat. Commun.* **12**, 1973 (2021).

55. J. Li, Y. Tong, L. Guan, S. Wu, D. Li, A UV-visible absorption spectrum denoising method based on EEMD and an improved universal threshold filter. *RSC Adv.* **8**, 8558–8568 (2018).

56. S. Hao, S. Suebka, J. Su, Single 5-nm quantum dot detection via microtoroid optical resonator photothermal microscopy. *Light Sci. Appl.* **13**, 195 (2024).

57. R. Houdré, R. Stanley, M. Ilegems, Vacuum-field Rabi splitting in the presence of inhomogeneous broadening: Resolution of a homogeneous linewidth in an inhomogeneously broadened system. *Phys. Rev. A* **53**, 2711–2715 (1996).

58. J. Yuen-Zhou, S. K. Saikin, V. M. Menon, Molecular emission near metal interfaces: The polaritonic regime. *J. Phys. Chem. Lett.* **9**, 6511–6516 (2018).

59. D. J. Tannor, R. Kosloff, S. A. Rice, Coherent pulse sequence induced control of selectivity of reactions: Exact quantum mechanical calculations. *J. Chem. Phys.* **85**, 5805–5820 (1986).

60. E. J. Heller, Y. Yang, L. Kocia, W. Chen, S. Fang, M. Borunda, E. Kaxiras, Theory of graphene Raman scattering. *ACS Nano* **10**, 2803–2818 (2016).

61. M. Osawa, N. Matsuda, K. Yoshii, I. Uchida, Charge transfer resonance Raman process in surface-enhanced Raman scattering from p-aminothiophenol adsorbed on silver: Herzberg-Teller contribution. *J. Phys. Chem.* **98**, 12702–12707 (1994).

62. S. C. Dong Wei, Q. Liu, Review of fluorescence suppression techniques in Raman spectroscopy. *Appl. Spectr. Rev.* **50**, 387–406 (2015).

63. C. He, Y. Wang, C. Waldfried, G. Yang, J. F. Zheng, S. Hu, H. X. Tang, Ultra-high Q alumina optical microresonators in the UV and blue bands. *Opt. Express* **31**, 33923–33929 (2023).

64. X. Liu, A. W. Bruch, Z. Gong, J. Lu, J. B. Surya, L. Zhang, J. Wang, J. Yan, H. X. Tang, Ultra-high-Q UV microring resonators based on a single-crystalline AlN platform. *Optica* **5**, 1279–1282 (2018).

65. G. Perin, Y. Dumeige, P. Féron, S. Trebaol, High-Q whispering-gallery-modes microresonators in the near-ultraviolet spectral range. arXiv:2211.03391 [physics.optics] (2022), https://arxiv.org/abs/2211.03391.

66. K. D. Heylman, N. Thakkar, E. H. Horak, A. M. Nelson-Quillin, C. Cherqui, K. A. Knapper, D. J. Masiello, R. H. Goldsmith, Optical microresonators as single-particle absorption spectrometers. *Nat. Photon.* **10**, 788–795 (2016).

67. M. Tse, H. Yu, N. Kijbunchoo, A. Fernandez-Galiana, P. Dupej, L. Barsotti, C. D. Blair, D. D. Brown, S. E. Dwyer, A. Effler, M. Evans, P. Fritschel, V. V. Frolov, A. C. Green, G. L. Mansell, F. Matichard, N. Mavalvala, D. E. McClelland, L. McCuller, T. McRae, J. Miller, A. Mullavey, E. Oelker, I. Y. Phinney, D. Sigg, B. J. J. Slagmolen, T. Vo, R. L. Ward, C. Whittle, R. Abbott, C. Adams, R. X. Adhikari, A. Ananyeva, S. Appert, K. Arai, J. S. Areeda, Y. Asali, S. M. Aston, C. Austin, A. M. Baer, M. Ball, S. W. Ballmer, S. Banagiri, D. Barker, J. Bartlett, B. K. Berger, J. Betzwieser, D. Bhattacharjee, G. Billingsley, S. Biscans, R. M. Blair, N. Bode, P. Booker, R. Bork, A. Bramley, A. F. Brooks, A. Buikema, C. Cahillane, K. C. Cannon, X. Chen, A. A. Ciobanu, F. Clara, S. J. Cooper, K. R. Corley, S. T. Countryman, P. B. Covas, D. C. Coyne, L. E. H. Datrier, D. Davis, C. di Fronzo, J. C. Driggers, T. Etzel, T. M. Evans, J. Feicht, P. Fulda, M. Fyffe, J. A. Giaime, K. D. Giardina, P. Godwin, E. Goetz, S. Gras, C. Gray, R. Gray, A. Gupta, E. K. Gustafson, R. Gustafson, J. Hanks, J. Hanson, T. Hardwick, R. K. Hasskew, M. C. Heintze, A. F. Helmling-Cornell, N. A. Holland, J. D. Jones, S. Kandhasamy, S. Karki, M. Kasprzack, K. Kawabe, P. J. King, J. S. Kissel, R. Kumar, M. Landry, B. B. Lane, B. Lantz, M. Laxen, Y. K. Lecoeuche, J. Leviton, J. Liu, M. Lormand, A. P. Lundgren, R. Macas, M. MacInnis, D. M. Macleod, S. Márka, Z. Márka, D. V. Martynov, K. Mason, T. J. Massinger, R. McCarthy, S. McCormick, J. McIver, G. Mendell, K. Merfeld, E. L. Merilh, F. Meylahn, T. Mistry, R. Mittleman, G. Moreno, C. M. Mow-Lowry, S. Mozzon, T. J. N. Nelson, P. Nguyen, L. K. Nuttall, J. Oberling, R. J. Oram, B. O'Reilly, C. Osthelder, D. J. Ottaway, H. Overmier, J. R. Palamos, W. Parker, E. Payne, A. Pele, C. J. Perez, M. Pirello, H. Radkins, K. E. Ramirez, J. W. Richardson, K. Riles, N. A. Robertson, J. G. Rollins, C. L. Romel, J. H. Romie, M. P. Ross, K. Ryan, T. Sadecki, E. J. Sanchez, L. E. Sanchez, T. R. Saravanan, R. L. Savage, D. Schaetzl, R. Schnabel, R. M. S. Schofield, E. Schwartz, D. Sellers, T. J. Shaffer, J. R. Smith, S. Soni, B. Sorazu, A. P. Spencer, K. A. Strain, L. Sun, M. J. Szczepańczyk, M. Thomas, P. Thomas, K. A. Thorne, K. Toland, C. I. Torrie, G.

Traylor, A. L. Urban, G. Vajente, G. Valdes, D. C. Vander-Hyde, P. J. Veitch, K. Venkateswara, G. Venugopalan, A. D. Viets, C. Vorvick, M. Wade, J. Warner, B. Weaver, R. Weiss, B. Willke, C. C. Wipf, L. Xiao, H. Yamamoto, M. J. Yap, H. Yu, L. Zhang, M. E. Zucker, J. Zweizig, Quantum-enhanced advanced LIGO detectors in the era of gravitational-wave astronomy. *Phys. Rev. Lett.* **123**, 231107 (2019).

68. V. Giovannetti, S. Lloyd, L. Maccone, Advances in quantum metrology. *Nat. Photon.* **5**, 222–229 (2011).

69. A. Pizzi, A. Gorlach, N. Rivera, A. Nunnenkamp, I. Kaminer, Light emission from strongly driven many-body systems. *Nat. Phys.* **19**, 551–561 (2023).

70. K. Murase, R. Laha, S. Ando, M. Ahlers, Testing the dark matter scenario for PeV neutrinos observed in IceCube. *Phys. Rev. Lett.* **115**, 071301 (2015).

71. Y. Fukuda, T. Hayakawa, E. Ichihara, K. Inoue, K. Ishihara, H. Ishino, Y. Itow, T. Kajita, J. Kameda, S. Kasuga, K. Kobayashi, Y. Kobayashi, Y. Koshio, M. Miura, M. Nakahata, S. Nakayama, A. Okada, K. Okumura, N. Sakurai, M. Shiozawa, Y. Suzuki, Y. Takeuchi, Y. Totsuka, S. Yamada, M. Earl, A. Habig, E. Kearns, M. D. Messier, K. Scholberg, J. L. Stone, L. R. Sulak, C. W. Walter, M. Goldhaber, T. Barszczxak, D. Casper, W. Gajewski, P. G. Halverson, J. Hsu, W. R. Kropp, L. R. Price, F. Reines, M. Smy, H. W. Sobel, M. R. Vagins, K. S. Ganezer, W. E. Keig, R. W. Ellsworth, S. Tasaka, J. W. Flanagan, A. Kibayashi, J. G. Learned, S. Matsuno, V. J. Stenger, D. Takemori, T. Ishii, J. Kanzaki, T. Kobayashi, S. Mine, K. Nakamura, K. Nishikawa, Y. Oyama, A. Sakai, M. Sakuda, O. Sasaki, S. Echigo, M. Kohama, A. T. Suzuki, T. J. Haines, E. Blaufuss, B. K. Kim, R. Sanford, R. Svoboda, M. L. Chen, Z. Conner, J. A. Goodman, G. W. Sullivan, J. Hill, C. K. Jung, K. Martens, C. Mauger, C. M. Grew, E. Sharkey, B. Viren, C. Yanagisawa, W. Doki, K. Miyano, H. Okazawa, C. Saji, M. Takahata, Y. Nagashima, M. Takita, T. Yamaguchi, M. Yoshida, S. B. Kim, M. Etoh, K. Fujita, A. Hasegawa, T. Hasegawa, S. Hatakeyama, T. Iwamoto, M. Koga, T. Maruyama, H. Ogawa, J. Shirai, A. Suzuki, F. Tsushima, M. Koshiba, M. Nemoto, K. Nishijima, T. Futagami, Y. Hayato, Y. Kanaya, K. Kaneyuki, Y. Watanabe, D. Kielczewska, R. A. Doyle, J. S. George, A. L. Stachyra, L. L. Wai, R. J. Wilkes, K. K. Young, Evidence for oscillation of atmospheric neutrinos. *Phys. Rev. Lett.* **81**, 1562–1567 (1998).

72. K. S. Thorne, Gravitational waves. arXiv:gr-qc/9506086 (1995), https://doi.org/10.48550/arXiv.gr-qc/9506086.

73. S. Higashino, Y. Mori, Y. Takubo, T. Higuchi, A. Ishikawa, I. Tsutsui, Weak value amplification in high energy physics: A case study for precision measurement of *CP* violation in *B* meson decays. *Phys. Rev. D* **104**, 033001 (2021).

74. J. R. Batley, R. S. Dosanjh, T. J. Gershon, G. E. Kalmus, C. Lazzeroni, D. J. Munday, E. Olaiya, M. Patel, M. A. Parker, T. O. White, S. A. Wotton, R. Arcidiacono, G. Barr, G. Bocquet, A. Ceccucci, T. Cuhadar-Dönszelmann, D. Cundy, N. Doble, V. Falaleev, L. Gatignon, A. Gonidec, B. Gorini, P. Grafström, W. Kubischta, I. Mikulec, A. Norton, S. Palestini, B. Panzer-Steindel, D. Schinzel, H. Wahl, C. Cheshkov, P. Hristov, V. Kekelidze, D. Madigojine, N. Molokanova, Y. Potrebenikov, A. Zinchenko, P. Rubin, R. Sacco, A. Walker, D. Bettoni, R. Calabrese, P. Dalpiaz, J. Duclos, P. L. Frabetti, A. Gianoli, M. Martini, L. Masetti, F. Petrucci, M. Savrié, M. Scarpa, A. Bizzeti, M. Calvetti, G. Collazuol, E. Iacopini, M. Lenti, F. Martelli, G. Ruggiero, M. Veltri, D. Coward, M. Eppard, A. Hirstius, K. Holtz, K. Kleinknecht, U. Koch, L. Köpke, P. Lopes da Silva, P. Marouelli, I. Mestvirishvili, C. Morales, I. Pellmann, A. Peters, B. Renk, S. A. Schmidt, V. Schönharting, R. Wanke, A. Winhart, J. C. Chollet, L. Fayard, G. Graziani, L. Iconomidou-Fayard, G. Unal, I. Wingerter-Seez, G. Anzivino, P. Cenci, E. Imbergamo, G. Lamanna, P. Lubrano, A. Mestvirishvili, A. Nappi, M. Pepe, M. Piccini, M. Valdata-Nappi, R. Casali, C. Cerri, M. Cirilli, F. Costantini, R. Fantechi, L. Fiorini, S. Giudici, I. Mannelli, G. Pierazzini, M. Sozzi, J. B. Cheze, M. de Beer, P. Debu, F. Derue, A. Formica, G. Gouge, G. Marel, E. Mazzucato, B. Peyaud, R. Turlay, B. Vallage, M. Holder, A. Maier, M. Ziolkowski, C. Biino, N. Cartiglia, M. Clemencic, F. Marchetto, E. Menichetti, N. Pastrone, J. Nassalski, E. Rondio, W. Wislicki, S. Wronka, H. Dibon, M. Jeitler, M. Markytan, G. Neuhofer, M. Pernicka, A. Taurok, L. Widhalm, A precision measurement of direct CP violation in the decay of neutral kaons into two pions. *Phys. Lett. B* **544**, 97–112 (2002).

75. B. J. Daigle, M. K. Roh, D. T. Gillespie, L. R. Petzold, Automated estimation of rare event probabilities in biochemical systems. *J. Chem. Phys.* **134**, 044110 (2011).

76. S. Suebka, E. McLeod, J. Su, Ultra-high-Q free-space coupling to microtoroid resonators. *Light Sci. Appl.* **13**, 75 (2024).

77. K. A. Knapper, E. H. Horak, C. H. Vollbrecht, K. D. Heylman, R. H. Goldsmith, Phase-sensitive photothermal imaging of ultrahigh-Q polyoxide toroidal microresonators. *Appl. Phys. Lett.* **113**, (2018).

78. L. Giannelli, E. Paladino, M. Grajcar, G. S. Paraoanu, G. Falci, Detecting virtual photons in ultrastrongly coupled superconducting quantum circuits. *Phys. Rev. Res.* **6**, 013008 (2024).

79. P. Forn-Díaz, L. Lamata, E. Rico, J. Kono, E. Solano, Ultrastrong coupling regimes of light-matter interaction. *Rev. Mod. Phys.* **91**, 025005 (2019).

80. A. Tokmakoff, “Nonlinear and two-dimensional spectroscopy notes” (2011), https://tdqms.uchicago.edu/2d-spectroscopy-notes/.

81. P. An, C.-Q. Yuan, X.-H. Liu, D.-B. Xiao, Z.-X. Luo, Vibrational spectroscopic identification of isoprene, pinenes and their mixture. *Chin. Chem. Lett.* **27**, 527–534 (2016).
